# The danger of not being typical: The positive effect of webpage prototypicality on users’ attitudes

**DOI:** 10.1007/s12525-025-00777-9

**Published:** 2025-05-14

**Authors:** Kathrin Figl, Aliaksei Miniukovich, Christiane Ernst, Christiane Lehrer

**Affiliations:** 1 Department of Information Systems, Production and Logistics Management, University of Innsbruck, Innrain 52, 6020 Innsbruck, Austria; 2 Department of Design, Norwegian University of Science and Technology, Raufossvegen 40, Entrance A, 2821 Gjøvik, Norway; 3 Department of Digitalization, Copenhagen Business School, Howitzvej 60, 2000 Frederiksberg, Denmark

**Keywords:** Prototype theory, Prototypicality, Elaboration likelihood model, Web design, Experiment, M31, C90, D83, O33, L86

## Abstract

In today’s competitive digital economy, effective web design is crucial for shaping user perceptions of organizations. While prior research has primarily focused on a single or few low-level design variables—such as color choices or banner layouts—this study explores webpage prototypicality—the extent to which a webpage aligns with typical design conventions in its genre—as an overarching design variable that encompasses various sub-variables (e.g., navigation structure, color schemes, and layout organization). Drawing on the elaboration likelihood model, we explore how webpage prototypicality interacts with message strength and users’ cognitive elaboration to affect users’ attitudes toward an organization. Through an online experiment on commercial banking websites, we found that higher prototypicality significantly improves users’ attitudes, specifically their general attitude towards the banks and the banks’ attractiveness as employers, highlighting its role as an effective design variable. Furthermore, our study shows that prototypicality plays a mediating role in the effects of message strength on users’ attitudes. This research contributes to research on web design in the fields of information systems and human–computer interaction and provides guidance for effective web design practices.

**Supplementary Information:**

The online version contains supplementary material available at 10.1007/s12525-025-00777-9.

## Introduction

In today’s digital economy, where businesses compete vigorously for attention, professional web design is no longer a luxury, but a necessity. Websites serve as critical touchpoints for diverse stakeholders, shaping their perceptions of the organization (Liu & Goodhue, 2012). This significant influence has stimulated extensive research in information systems (IS), focusing on the impact of various design variables, including colors (Pelet & Papadopoulou, 2012), third-party seals (Hassna et al., 2023), and the presence of advertisements (Werner et al., 2022). While these studies provide valuable insights into individual or a few design variables, they fail to capture the collective influence of these variables when considered as a cohesive whole. Although we do not question the achievements of prior research, our study emphasizes the importance of a broader view in web design. We aim to explore webpage prototypicality as an overarching design variable that encompasses various sub-variables, thereby advancing our understanding of the holistic impact of web design.

Prototypicality refers to the degree to which an item is perceived to represent a particular category (e.g., Loken & Ward, 1990; Quintal & Phau, 2013); in our context, webpage prototypicality refers to the degree to which a webpage is perceived to represent a class of websites (e.g., Miniukovich et al., 2024). For instance, a highly prototypical banking website typically features a clean layout, a prominent logo in the top corner, a central banner image, clear navigation menus, and consistent use of corporate colors. Such design choices align with user expectations and established design conventions within the industry. Research indicates that individuals subconsciously prefer high prototypicality (Reber et al., 2004). This preference is often associated with better evaluations, as demonstrated in studies on company brands (Kalamas et al., 2006; Quintal & Phau, 2013), product package designs (Celhay & Trinquecoste, 2015; van Ooijen et al., 2016), and retail stores (Babin & Babin, 2001). For businesses offering digital touchpoints, adopting prototypical design could effectively enhance users’ attitudes.

**Fig. 1:**
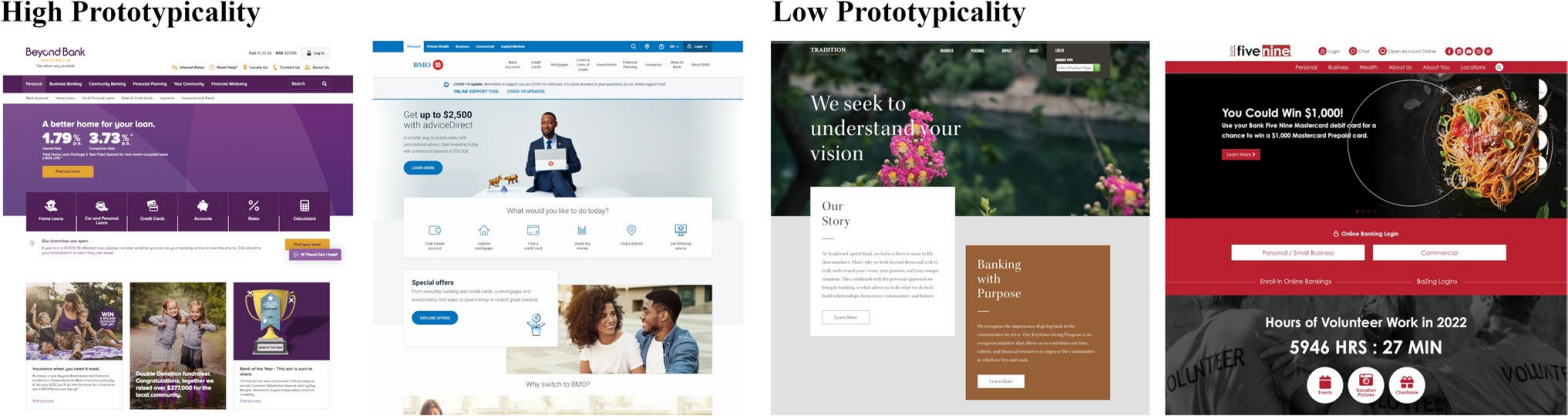
Real-world examples of high (left side) and low (right side) prototypicality homepages of commercial banks

Figure 1 illustrates the concept of prototypicality in web design with real-world examples of banking homepages with varying levels of prototypicality. In a highly digitalized banking landscape, websites serve as primary contact points. Given the high degree of commoditization of banking products and services, some banks try to differentiate themselves through innovative web design (see Fig. 1). However, the response of users to prototypical versus atypical web design remains unclear.

Previous studies on webpage prototypicality indicate that highly prototypical webpages are perceived more positively in terms of appeal (Tuch et al., 2012) and trustworthiness (Miniukovich & Figl, 2023). However, existing studies have notable limitations, as they often rely on correlational data (e.g., Miniukovich & Figl, 2023), treat prototypicality only as a secondary variable (e.g.,Tella, 2019), or focus primarily on aesthetic outcomes rather than practically relevant measures such as user attitudes and preferences. These limitations underscore the need for more comprehensive and methodologically robust approaches to studying prototypicality—an increasingly urgent task in light of recent technological developments. The rapid advancement and accessibility of AI-driven web design tools have transformed the landscape of modern web design, enabling the creation of more diverse and highly customized webpage layouts with minimal effort (see e.g., Muthazhagu & Surendiran, 2024). These tools facilitate greater experimentation with design variables, underscoring the importance of understanding the role of prototypicality. Moreover, a recent study has identified specific brain areas and neural pathways involved in how prototypicality as a design variable influences user design preferences, distinguishing it from other design variables such as simplicity and emphasizing the relevance of prototypicality (Cho et al., 2024).

Backed up by these different advancements, our study seeks to extend prior research by systematically analyzing the causal relationship between webpage prototypicality and user attitudes, moving beyond correlational insights. By examining user responses to prototypical versus atypical webpage designs, this research not only enhances our theoretical understanding of digital touchpoint interactions but also provides actionable insights for interface design. Such insights are valuable for businesses seeking to optimize user experience and improve conversion rates across their digital touchpoints. Thus, we ask:

- **RQ1**: Does webpage prototypicality positively affect users’ attitudes toward an organization?

In this paper, we employ the elaboration likelihood model (ELM) (Petty & Cacioppo, 1986) from social psychology as our theoretical framework. In line with the ELM, we conceptualize a website as an environment providing diverse stimuli, encompassing both visual cues (i.e., prototypical design) and textual cues (Tam & Ho, 2005). When such stimuli attract the user’s attention and are mentally processed, they can influence the user’s attitude toward the target entity. Websites, essentially, serve as persuasive tools designed to align with a company’s objectives, aiming to positively influence users’ perceptions, including their attitude toward the company represented on the website (Cyr et al., 2018).

According to the ELM, users form attitudes through two distinct routes: the peripheral route, influenced by aspects like visual design (peripheral cues), and the central route, influenced by the strength of argumentation in a textual message (central cues). The choice of route depends on individuals’ “elaboration likelihood,” i.e., how deeply they engage with information. In web interactions users tend to behave as “cognitive misers” (Liu & Goodhue, 2012), reducing their cognitive effort in engaging deeply with textual information and favoring the peripheral route. This further emphasizes the potential role of webpage prototypicality in attitude formation. While the main focus of this study is on webpage prototypicality, incorporating message strength as a complementary factor allows us to assess the interplay between visual and textual elements in influencing user attitudes. Moreover, by considering different levels of cognitive elaboration, our study provides a more nuanced view of how webpage prototypicality and message strength shape user attitudes within digital environments. This leads us to our second research question:

- **RQ2**: How does users’ cognitive elaboration moderate the relationships between webpage prototypicality, message strength, and users’ attitudes toward an organization?

To address these two research questions, we conducted an online study involving commercial banking websites. We focused on homepages, which are the entry point for users visiting a website and play “a pivotal role in either luring consumers into a Web site or in driving them away” (Geissler et al., 2001, p. 2). In this study, webpage prototypicality served as a predictor of attitude toward the bank, particularly regarding perceptions of the bank as a potential employer. By investigating how prototypicality influences user attitudes, this study aims to contribute to research on web design within the field of IS, while also providing valuable practical insights for website designers and AI-driven web design processes.

## Theoretical background

### Prior research on web design

A considerable body of IS research addresses the effects of web design on users. Within the Senior Scholars’ List of Premier IS Journals,^1^ a plethora of publications have explored multifaceted aspects of web design. Frequently investigated dependent variables include users’ intention to use (Agarwal & Karahanna, 2000; Jahng et al., 2007; Pelet & Papadopoulou, 2012; Visinescu et al., 2015), usability (Agarwal & Venkatesh, 2002; Chen, 2018), satisfaction (Ho et al., 2011; Zviran et al., 2006), and trust (White Baker et al., 2019). These studies also cover a broad range of independent variables, examining such factors as ease of use (Agarwal & Venkatesh, 2002; White Baker et al., 2019), website type (Ho et al., 2011; Jin et al., 2023), interaction richness (Singh et al., 2005) and familiarity (Lowry et al., 2008; Sia et al., 2009), as well as specific design variables, like images (Winter et al., 2003), content (Geissler et al., 2001), and the use of animation (Geissler et al., 2001), as well as aesthetic design variables like visual complexity (Chen, 2018; Deng & Poole, 2010) and colors (Pelet & Papadopoulou, 2012). Notably, studies by White Baker et al. (2019) and Jin et al. (2023) have shed light on user attitudes in varying digital environments, laying the groundwork for examining how these attitudes develop within particular contexts. Moreover, Wang et al. (2009) showed how congruity between a brand’s online and offline presence influences users’ reliance on attitude transfer, as opposed to piecemeal processing. This congruity reinforces consumer expectations and leads to more positive attitudes, supporting the idea that web design can facilitate heuristic processing through familiar structures. Additionally, Ye et al. (2020) demonstrated the importance of visual aesthetics for the formation of webpage impression. The aesthetic dimensions of visual complexity, unity, and novelty are important design variables that influence attitude formation.

This robust body of work underlines that web design plays a central role in shaping user attitudes. However, it tends to focus on specific design variables of a webpage, emphasizing the need to examine broader constructs that may unify design features and clarify their influence on user attitudes.

### Prototypicality

Prototypicality offers one such unifying construct that addresses this broader perspective on web design. Originating from information-processing psychology (Rosch & Lloyd, 1978), it explains how humans categorize objects, ideas, and concepts they encounter. Prototypicality is defined as “the amount to which an object is representative of a class of objects” (Leder et al., 2004, p. 496). Thus, a prototype is the most typical or salient member of a category (Rosch & Mervis, 1975). For example, a robin or a sparrow may be most often associated with the category “bird,” but a penguin may not. Other items are included in the category based on how closely their attributes align with the prototype (Rosch & Mervis, 1975). Individuals are faster in identifying prototypes and name prototypes more frequently when asked to give examples in a category (Rosch, 1973). Moreover, empirical studies in cognitive psychology have shown that individuals favor prototypical exemplars over non-prototypical ones. In the context of websites, Bellman and Rossiter (2004) introduced the concept of the “website schema,” emphasizing that congruence between a user’s mental model and the actual structure of a website improves navigability and positively affects user attitudes. Their findings align with prototypicality research, indicating that users respond more favorably when websites match their expectations for layout and functionality. Additionally, previous research has shown that prototypicality in advertisements reduces the depth of cognitive engagement, as individuals rely on pre-existing category schemas to process the content (Goodstein, 1993). Building on this, we examine how webpage prototypicality influences users’ attitudes toward organizations. In a manner similar to advertisements, websites often function as visual representations of a brand, where prototypicality may act as a peripheral cue, shaping user perceptions with minimal cognitive effort.

Prototype theory challenges classical categorization theories (Laurence & Margolis, 1999), which posit that categories are defined by a distinct and necessary set of features that all members of the category possess (Rosch & Mervis, 1975). According to prototype theory, there may be no single feature that all category members share (Geeraerts, 1989). Instead, members of a category are related to each other through a network of shared features or characteristics, even though they may not all share the same defining features. These shared features contribute to the overall perception that these members belong to the same category. An analogy can be drawn to how members of a family can share certain physical or behavioral traits without all being identical or having the same set of characteristics (Cantor & Mischel, 1979). Within a category, the prototype represents the most typical or central example of the category, and other members are judged in relation to it. Some members may exhibit a stronger resemblance to the prototype, while others may exhibit a weaker resemblance (Rosch & Mervis, 1975). For example, an apple might be seen as a more prototypical fruit than a dragon fruit.

Subjective experiences and learning play significant roles in shaping how individuals develop and refine their category prototypes (Mervis & Rosch, 1981). Individuals’ subjective experiences, such as personal encounters with category members or cultural influences, can impact the formation of their category prototypes (Mervis & Rosch, 1981). Through learning, individuals become more adept at recognizing category members and understanding the diversity of features that can be associated with a category (Loken & Ward, 1990). For example, users know what a typical bank homepage looks like, not because they have read a definition of the commercial banking category, but because they have seen several bank webpages multiple times. Thus, the webpages seen contribute to the users’ idea of commercial banking webpages, with the contribution of each webpage depending on the frequency of visiting that webpage. This frequency is referred to as the frequency of instantiation and is a predictor of prototypicality (Loken & Ward, 1990). Consequently, prototypicality studies must account for each user’s familiarity with a particular website or brand, as familiarity can directly influence prototypicality or affect the user attitudes under investigation (e.g., trust; Kuen et al. (2023).

Empirical studies across domains have consistently shown that individuals prefer prototypical designs over atypical ones, for example, in car design (Kazinnik & Curti, 2022; Mayer & Landwehr, 2018) and product aesthetics (Hekkert et al., 2003; Veryzer Jr & Hutchinson, 1998). This preference for typicality extends to stimulus domains like human faces (Langlois & Roggman, 1990) and abstract visual patterns (Winkielman et al., 2006). Notably, prototypicality also plays a crucial role in consumers’ perception of product trendiness and the evaluation of fit between a product and its presentation context (Blijlevens et al., 2013).

The few studies that specifically explored webpage prototypicality focused on the relationships between webpage prototypicality and aesthetics (Tuch et al., 2012), trust (Miniukovich & Figl, 2023), or task-solving performance (Roth et al., 2013). These studies found that webpages with high prototypicality were perceived as more appealing. Few studies have explicitly included prototypicality as a predictor of users’ first impression, preference, or attitude. A notable exception is a recent study by Miniukovich and Figl (2023). Based on crowdworker evaluation of over 3000 websites, the results showed a strong direct positive effect of prototypicality on the trustworthiness of webpages, stronger than effects of usability and webpage aesthetics. The study, however, provided only correlational data, making causal inferences difficult. Other studies on webpage prototypicality (Douneva et al., 2016; Tella, 2019; Tuch et al., 2012; Ye et al., 2020) also have limitations, such as treating prototypicality only as a confounder, including very few stimuli, not manipulating prototypicality systematically, or focusing on webpage aesthetics as the outcome instead of more practically useful variables such as user attitudes. Investigating prototypicality as a more holistic web design concept may therefore yield new insights into how consistent layout structures shape user attitudes and behaviors. More specifically, our study aims to extend existing research by systematically investigating the causal impact of webpage prototypicality on user attitudes.

### The elaboration likelihood model

An important theoretical framework that clarifies why prototypicality can influence user attitudes is the ELM of persuasion (Petty, 1981; Petty & Cacioppo, 1986), which offers a theoretical explanation of how individuals process stimuli in different contexts. In IS research, the model has been applied to investigate the persuasiveness of website elements such as text, images, or videos in various contexts, including online reviews (Qahri-Saremi & Montazemi, 2025), web personalization (Ho & Bodoff, 2014; Tam & Ho, 2005), social media (Cummings & Dennis, 2018; Shin et al., 2020), and crowdfunding (Wang et al., 2021). Peterson and Malhotra (2023) offer additional insights into how typicality judgments affect the processing of creative advertisements, demonstrating that highly typical ads can facilitate easier cognitive processing through alignment with consumer expectations.

The ELM, a dual process theory, distinguishes between two cognitive routes, which can both result in attitude formation or change: the *central* and the *peripheral route*. These two routes are distinct in the depth of cognitive processing, the types of information processed, and the persistence of attitude change (Bhattacherjee & Sanford, 2006). In the central route, individuals engage in extensive cognitive processing, carefully analyzing information presented (e.g., the logic of the arguments). Elaboration involves attending to the content as well as examining and evaluating the quality and strength of presented arguments (Petty & Cacioppo, 1986). In contrast, the peripheral route involves less intensive cognitive processing, relying on heuristics and situational cues (Petty, 2013). Attitudes formed via the central route tend to be more persistent than those formed via the peripheral route.

The likelihood of cognitive effort an individual will allocate to processing a message (*elaboration likelihood*) depends on their *motivation* (the desire to process information) and *ability* (the capability for critical evaluation), both of which should be present for extensive elaboration to occur (Petty & Cacioppo, 1986). While motivation results, for example, from the personal relevance of the issue at hand, ability is affected by aspects such as distractions, cognitive load, and prior knowledge (Schumann et al., 2012). It should be noted that elaboration likelihood is not a stable personality characteristic, but rather a temporary state influenced by situational contexts and time, even for the same individual (Bhattacherjee & Sanford, 2006). When individuals are highly motivated and capable of thinking about a message, the arguments presented become paramount. They tend to follow the central route by critically examining the information and focusing on its factual merits. In the context of web design, this suggests that users who are highly motivated and have the ability to process information will thoroughly analyze and evaluate the website’s content and the strength of the presented arguments (Cyr et al., 2018), known as *message strength* (Petty & Cacioppo, 1984). This careful consideration leads to deeper cognitive processing, which in turn increases attitude persistence.

In contrast, when motivation or ability to evaluate message arguments is lacking, for example, due to lower personal relevance of the information or distractions, an individual’s attitude can still be influenced by other variables, serving as peripheral cues in the communication environment. Peripheral cues are superficial or non-core elements in the communication environment, often tangential to the primary content (Petty & Cacioppo, 1986). In this case, individuals follow the peripheral route and apply simple heuristics (i.e., rules of thumb). In the context of web design, peripheral cues refer to quickly and easily perceivable features of a website (San José Cabezudo et al., 2009). They indirectly signal to individuals that the product or company is likely to meet their expectations, resembling the “halo” effect (Hartmann et al., 2008)—a subconscious association of one aspect with another (e.g., “This is a prototypical website, so the company should be trustworthy.”) Multiple website features have been identified as factors that serve as peripheral cues, including webpage image appeal and navigation design (Cyr et al., 2018), font types and color design (Pelet & Papadopoulou, 2012; San José Cabezudo et al., 2009), and overall visual webpage aesthetics (Tractinsky & Lowengart, 2007). Although these studies demonstrate the empirical validity of the ELM in the context of web design, none of them examined the influence of prototypicality. Our research emphasizes prototypicality as a potential peripheral cue (Kitchen et al., 2014). Notably, studies in the context of product design highlight the significance of prototypicality as a peripheral cue, demonstrating its comparable importance to message strength (van Ooijen et al., 2016).

Taken together, these observations suggest that prototypical design features function as influential peripheral cues under low-elaboration conditions, thereby exerting a meaningful impact on user attitudes.

## Research model and hypotheses development

**Fig. 2:**
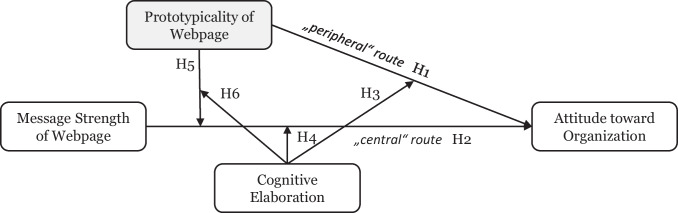
Research model

Drawing on the ELM, we argue that users form attitudes toward organizations represented on websites by considering both the website’s overall visual presentation (i.e., prototypicality) and the strength of the presented arguments (i.e., message strength), as shown in Fig. 2. Prototypicality is expected to serve as an effective peripheral cue (Cyr et al., 2018), positively influencing user attitudes (Samson & Voyer, 2012; San José Cabezudo et al., 2009). In addition, message strength is included as an independent variable, reflecting its established role within the central route to persuasion (Cyr et al., 2018; van Ooijen et al., 2016). We further propose that the extent of cognitive elaboration by the user (Petty & Wegener, 1999) moderates the effects of both webpage prototypicality and message strength on attitudes toward an organization. Moreover, our research model suggests that cognitive elaboration and webpage prototypicality interact to moderate the effect of message strength on users’ attitudes toward an organization. Specifically, the moderating effect of low prototypicality is amplified when cognitive elaboration is low. We detail our reasoning in the following sections as we develop the hypotheses.

### Relationship between prototypicality and attitude

An attitude refers to an individual’s psychological tendency to evaluate a particular entity with varying degrees of favor or disfavor (Eagly & Chaiken, 1993). Attitudes develop and adapt as individuals gain and process information about these entities (Eagly & Chaiken, 1993). In marketing research, a distinction is made between consumers’ attitudes toward marketing stimuli (e.g., an ad), and their response to the brand represented by these stimuli (Najmi et al., 2012). In this study, we focus on examining how both prototypicality and message strength influence users’ attitudes toward the latter—the organization represented by the website—rather than toward the website itself.

Previous research has shown that various webpage design variables, such as images, color, and text design, act as relevant peripheral cues affecting user attitude (Cyr et al., 2018; San José Cabezudo et al., 2009). We regard prototypicality as an overarching feature of website design and view it as a combination of other design variables that influence attitude formation via the peripheral route (North & Hargreaves, 2000). We anticipate that prototypicality will have a positive influence on attitude, since prototypicality research has shown that individuals tend to prefer prototypical representations (Rosch, 1973). In our context, a webpage closely aligned with users’ mental prototype of a typical or ideal webpage—regarding layout, design, or content organization—might activate a heuristic response, resulting in a positive attitude toward the represented organization. Hence, we hypothesize:

- **H1:** Webpage prototypicality positively affects users’ attitudes toward an organization.

### Relationship between message strength and attitude

The influence of argument quality on persuasion and attitude change has been consistently established in research. Argument quality refers to an individual’s perception that message arguments are strong and convincing (Petty & Cacioppo, 1986). Stronger arguments appeal to rational thinking, withstand counterarguments, and convey credibility, which leads to greater acceptance and belief compared to weaker messages. This, in turn, leads to a more profound impact on attitude formation toward the target object (Petty & Cacioppo, 1986; Petty, 1981; Petty et al., 1983).

One stream of IS research has examined how the quality of arguments influences attitudes toward specific IT systems, such as personalization agents (Ho & Bodoff, 2014), electronic health records (Angst & Agarwal, 2009), or document management systems (Bhattacherjee & Sanford, 2006). However, our study focuses on the impact of argument quality on users’ attitudes toward the organization behind an IT artifact (i.e., a website). In the context of e-commerce, Kim and Benbasat (2009) demonstrated that strong trust-assuring arguments positively affect users’ trust beliefs in an online store provider. Zhou et al. (2016) similarly confirmed the importance of argument quality in establishing trust with website providers during users’ initial interactions. Lowry et al. (2012) showed how the quality of arguments on a website influences users’ perceptions of privacy assurance by an online travel-booking provider, subsequently impacting their behavioral intentions, such as making purchases. Marketing research studies have demonstrated that ads containing strong arguments positively influence consumer attitudes toward a product or brand (Lord et al., 1995; Petty et al., 1983).

In our study, argument quality refers to the strength of the arguments used to convey the benefits of an organization through the written content of the website. Based on prior findings on argument quality and its relationship with attitude formation, we hypothesize the following:

- **H2:** Message strength positively affects users’ attitudes toward an organization.

### Moderating effect of elaboration likelihood

Based on the ELM, it is possible to determine the factors primarily responsible for attitude formation by knowing the extent of elaboration likelihood. Prior work suggests that both motivation and ability affect elaboration likelihood. In this study, our primary theoretical focus is on *ability*, conceptualized as the cognitive capability of a user to process a message (here, a website). We are particularly interested in the effect of diminished cognitive ability due to additional cognitive load, which may be induced by the distractions and multitasking typically experienced when browsing webpages online. In the following, we theorize about the moderating effect of elaboration likelihood on the various relationships in our research model.

#### The moderating effect of cognitive elaboration in the relationship between webpage prototypicality and attitude

According to the ELM, when elaboration is low, factors acting as peripheral cues are primarily responsible for attitude formation, whereas when elaboration is high, the strength of the message is primarily responsible. Therefore, we anticipate that in situations where individuals interact with a website and engage under conditions of high cognitive elaboration, they will focus on textual content and the strength of the arguments provided. As a result, prototypicality will only have a limited impact on attitude formation, similar to other visual design variables such as webpage aesthetics (Tractinsky & Lowengart, 2007). However, in scenarios where individuals engage under conditions of low cognitive elaboration (e.g., due to external distractions), we expect prototypicality to become more important and serve as a peripheral cue used as an easy-to-process signal for the quality of an organization (Petty & Wegener, 1999).

For peripheral processing to occur, a corresponding decision rule must be cognitively available and perceived as reliable (Eagly & Chaiken, 1993). For example, when encountering a website, individuals may adopt a more positive attitude if it aligns with prototypical standards and has no atypical features. Previous studies have consistently shown that people prefer prototypical objects (Winkielman et al., 2006). In this context, a user may rely on the mental shortcut “This website is typical and thus signals competence of the organization behind it,” which shapes their subsequent attitude toward the organization. This aligns with the reasoning that when “perceivers do not have adequate time to engage in further processing, then initial perceptions will become [final, persistent] webpage impressions directly” (Ye et al., 2020, p. 930), implying that the effect of webpage prototypicality would shift from being unimportant to being the primary factor affecting attitude, potentially stronger than message strength. Hence, we hypothesize the following:

- **H3:** Lower cognitive elaboration strengthens the effect of webpage prototypicality on users’ attitudes toward an organization.

#### The moderating effect of cognitive elaboration in the relationship between message strength and attitude

The ELM suggests that users exhibiting high cognitive elaboration tend to meticulously process website information, scrutinizing arguments and facts presented. When visiting a new website, they may want to learn about the organization and what it has to offer by reading, for example, the “About Us” description. This information is processed via the central route, where users distinguish between strong and weak arguments. Under such conditions, attitudes toward the organization are shaped primarily by content offering strong and convincing arguments as opposed to content with weak arguments (Petty & Cacioppo, 1986). In contrast, if users are unable to engage in thorough cognitive processing, e.g., due to distractions while interacting with the website, they tend to rely more on superficial aspects of the website (e.g., visual aspects). Under heuristic processing, strong arguments embedded in the text may be overlooked or not considered in depth and, therefore, do not have the same impact on users’ attitudes toward the organization as they do under high elaboration (Petty & Cacioppo, 1986). Therefore, we hypothesize:

- **H4:** Lower cognitive elaboration weakens the effect of message strength on users’ attitudes toward an organization.

#### The moderating effect of cognitive elaboration on the effect of prototypicality on the influence of message strength

Prototypicality may also influence the type of elaboration chosen (Goodstein, 1993), as a non-prototypical design may stand out so much that it raises doubts about the design’s credibility, leading to more careful consideration and resulting in the use of central processing (van Ooijen et al., 2016) and the disregard of peripheral cues. A study by van Ooijen et al. (2016) showed that atypical product packaging shapes amplify the effect of message strength. Thus, we expect that low prototypicality increases the likelihood that individuals will employ high-effort central elaboration, especially when additional external cognitive load is low. Such central elaboration would, in turn, direct user attention to details, such as textual descriptions, increasing the effect of message strength. Therefore, the impact of the message on attitude is hypothesized to be moderated by prototypicality, with a stronger influence observed when prototypicality is low compared to when it is high. Thus, we propose:

- **H5:** Lower prototypicality of a webpage increases the effect of message strength on users’ attitudes toward an organization.

Moreover, low elaboration might increase the moderating effect of prototypicality on the influence of message strength on attitude toward an organization. For instance, when the message is weak and users do not engage cognitively, it is expected that a negative attitude will emerge, particularly if the organization’s webpage is atypical. Conversely, a strong message may still evoke a positive attitude when the webpage is typical, as users can rely on peripheral cues suggesting message credibility—even if they are not actively scrutinizing its arguments under low cognitive elaboration. Hence, we propose:

- **H6:** Lower cognitive elaboration amplifies the negative moderating effect of lower prototypicality on the effect of message strength on users’ attitudes toward an organization.

These hypotheses shed light on the intricate interplay between prototypical design and message strength on websites, as well as elaboration levels, providing insights into their joint role in shaping user attitudes.

## Research method

To empirically test our hypotheses, we conducted a mixed-design online experiment. It included three factors, each with two levels: (1) level of elaboration (high and low, between-subjects), (2) webpage prototypicality (high and low, within-subjects), counter-balanced with (3) message strength (weak and strong, within-subjects). We used prototypicality and message strength as within-subjects factors to increase statistical power, as having the same participants rate stimuli across factor levels reduces variability in the ratings caused by uncontrolled individual factors (e.g., mood or lighting in the room). To enhance generalizability, multiple stimuli represented each level of the within-subjects factors (eight high and eight low prototypicality pages paired with eight weak and eight strong messages), minimizing the effects of individual-stimulus peculiarities (e.g., the topics mentioned in a message or subjects of images on a webpage). Webpage prototypicality was the factor that represented the effects of design as a peripheral cue on user attitudes, while message strength represented core arguments and their effect on user attitudes. The level of elaboration was kept as a between-subjects factor—where low elaboration for one stimulus could influence subsequent evaluations—and to reduce the risk of participants becoming aware of the manipulations.

### Experimental procedure

We chose commercial banking as our research context for three main reasons. First, banking websites are frequently used by a wide range of users, who regularly engage with their banks online for activities such as checking account balances, making transactions, or finding a specific piece of information. This regular interaction has led to a well-established baseline for what users expect from a typical banking website, making deviations from this prototype particularly noticeable and impactful. Such a context is ideal for studying how variations from the norm influence user attitudes. Second, it can be assumed that participants have the necessary knowledge to assess the quality of the arguments presented in combination with the website, rather than having to rely on peripheral cues only. This ensures that the findings reflect informed judgments, providing greater validity to the results. Third, online banking is a business domain characterized by a high degree of homogeneity, with many institutions offering similar products and services. This uniformity allows us to control for extraneous variables that could otherwise confound the results, such as differences in product offerings. Furthermore, the importance of stability and trust in banking cannot be overstated; a prototypical webpage design is not only familiar but also reinforces perceptions of trustworthiness and professionalism, which are critical in this field.

The experimental procedure consisted of three steps: (1) The participants read the experimental instructions; (2) we randomly assigned them to one of the two between-subjects conditions (i.e., high vs. low elaboration) and asked them to evaluate 16 banking webpages; and (3) the final part consisted of a questionnaire about controls and demographics.

In the first step, a vignette was used to immerse participants in a scenario in which they were seeking a job as an assistant manager in a bank. Vignettes, which provide written descriptions of realistic situations (Trevino, 1992), were chosen to place all participants in the same scenario, thereby minimizing potential contamination of participants’ perceptions (Cummings & Dennis, 2018). We chose the job search scenario to elicit *motivation* (i.e., personal relevance, Bhattacherjee and Sanford (2006)) in all participants to process the stimuli provided in the experiment. However, as we describe in detail later, we reduced the *ability* to process information in half of the study participants (i.e., the low elaboration group). The participants were also instructed to consider each of the presented banking webpages individually and to rate their preference for employment at each bank. They were then informed that the “Next” button on each of the following pages would only appear after 10 s. By setting a minimum duration, we ensured that the participants spent time viewing the provided information, instead of skipping it. In step two, each participant was presented with 16 webpages of banks, one after the other (eight with low prototypicality and eight with high prototypicality in random order) and asked to rate their attitude toward the bank as a whole and as a potential employer. Random presentation of webpages was necessary to control for serial position effects, as the order in which information is presented can significantly influence user evaluations, with evaluations being less extreme at the beginning compared to the end of an evaluation series (Zorn & Unkelbach, 2023). The randomly drawn “About Us” descriptions or employer reviews (half with a weak vs. strong message) were presented directly above the screenshot of the respective webpage, without styling. We also included six attention check questions following the evaluation of the banks asking for a specific response to the questionnaire item (e.g., “disagree”) to ensure that participants were paying attention to the questionnaire. The low elaboration group was confronted with a simultaneous working memory task, as described further below.

### Manipulation of the independent variables

#### Manipulation of cognitive elaboration

Since elaboration likelihood is a temporary state, it can be manipulated by modifying, for example, the degree of distraction (Bhattacherjee & Sanford, 2006). To manipulate the level of cognitive elaboration, we created two conditions: high elaboration and low elaboration. In the low elaboration condition, participants were engaged in a secondary working memory task concurrently with the survey, acting as a distractor to reduce cognitive resources available for processing messages and webpage stimuli. We used 4 × 4-matrix working memory tasks (Bago & De Neys, 2019), each displayed for 5 s. Following Trémolière et al. (2012), participants were instructed to memorize the position of five dots within the matrix (see Appendix E) and then reproduce them after viewing and evaluating a message and a webpage. We generated 16 workload tasks presented randomly before each of the 16 messages and webpage stimuli.

In contrast, the high elaboration condition ensured that participants encountered no distractions, allowing them to dedicate their full cognitive resources to the efficient processing of the presented information.

#### Manipulation of webpage prototypicality

The process of selecting webpage stimuli for the study involved three steps (further detailed in Appendix A). First, we collected an initial sample of 1.032 bank webpages. Using multiple strategies, including search engine queries and Wikipedia lists, we obtained the URLs of website homepages, as homepages are the webpages most representative of the overall website design and content. We then captured the screenshots of the homepages with a custom Firefox add-on after removing temporary webpage elements, such as pop-ups and notification banners. Second, 1.298 MTurk crowdworkers rated these 1.032 webpages on their prototypicality and other design-related dimensions as potential confounders (e.g., visual aesthetics and complexity). Each crowdworker rated 71 distinct webpages on a single dimension. Finally, we identified the distinct groups of high and low prototypicality, based on the crowdworker ratings. Within each group, we selected eight homepage screenshots for this study. Our selection process was designed to minimize correlations between prototypicality and potential confounding factors. Additionally, it involved excluding widely known banks, institutions that were not exclusively commercial, and those not based in the USA. Screenshots—and not live webpages—were used as stimuli to ensure uniformity in what participants were exposed to, as live webpages could have their content or design updated at any moment or show different content depending on a participant’s location or browsing history, which would introduce extra random variance in our data. In this study, the job-search scenario primarily required activities such as viewing and reading content, rather than extensive interactions with webpages (e.g., filling out forms or sending queries to a system). Screenshots can accommodate this just as well as actual webpages.

#### Manipulation of message strength

In line with other ELM studies, we developed strong and weak arguments for a bank’s quality to examine the influence of message strength. Similar to prior studies (Cyr et al., 2018; San José Cabezudo et al., 2009), we operationalized such arguments as brief textual descriptions of commercial banks, which we created ourselves and evaluated in a pre-test. The descriptions were either “About Us” statements or employees’ reviews of banks, and eight were initially constructed for high and low message strength, resulting in 32 messages. By utilizing both employer reviews and “About Us” statements—distinct communication types normally found on a bank’s webpage and crafted either by users or by the bank—we aim to enhance the generalizability of our study’s conclusions in a job-search context. When creating the messages, we had to make sure they fit each bank’s homepage. Thus, we avoided messages that would contradict the textual descriptions in the homepage screenshots or facts that participants might know about the banks. Brand names, personal names, place names were replaced with placeholders such as [Company name], [Person X], and [Region]. The average message length was 71.3 words, professionally proofread.

Given that message strength is not a universal measure but is context-specific (Schumann et al., 2012), we subjected the message set to evaluation by crowdworkers on MTurk. To minimize non-message effects, the messages were shown as pure text with no styling, images, or brand references and no time pressure. Each crowdworker rated 30 messages (with five rated twice, for quality control) on four items (“The arguments of this “About Us”/employer review message are [convincing/strong/informative]” and “This snippet sounds like a realistic “About Us”/employer review statement.”) using seven-point Likert scales. Only crowdworkers from English-speaking countries (i.e., the USA, the UK, Australia, New Zealand, Canada, and Ireland) were involved. An average session lasted 15 min, and workers were compensated with 1.6 USD after quality control. The responses of 24 crowdworkers were of sufficient quality and were retained, resulting in approximately ten ratings per stimulus.

We then calculated the mean strength of each message (mean of the items convincing, strong, informative). Based on the pre-test, the four weakest and strongest messages per category (“About Us,” employees’ reviews) were selected, resulting in 16 messages overall. The character counts of the final messages ranged from a minimum of 317 to a maximum of 465 (*M* = 395, SD = 48.5). Examples of these messages are shown in Appendix E.

### Dependent variables: questionnaire measures

We used validated scales from the literature for all constructs and measured the questionnaire items using seven-point Likert scales ranging from *completely disagree* (1) to *completely agree* (7). To measure the *general attitude toward a bank*, we used the “ability” scale of the trust belief items by Brengman and Karimov (2012), adapted for the banking context*.* This construct reflects the user’s belief about the competence, skills, and knowledge of the bank. Additionally, the *attractiveness of the bank as an employer* was measured by five items adapted from Ageeva et al. (2018). In our sample, both scales demonstrated high internal consistency, with a Cronbach’s alpha of 0.97 for each construct. This indicates that the items within each scale demonstrated high internal consistency and reliability.

To further establish the construct validity of these dependent variables, we conducted both exploratory and confirmatory factor analysis (see Appendix C). The exploratory factor analysis, performed using maximum likelihood extraction with oblique (Promax) rotation, revealed a clear two-factor solution accounting for 88% of the total variance. All items loaded strongly on their respective factors (*attractiveness of the bank as an employer* items: loadings ranging from 0.87 to 0.95 on factor 1; *general attitude toward the bank* items: loadings ranging from 0.84 to 0.95 on factor 2) with no cross-loadings exceeding 0.32, thereby supporting the distinctiveness of the employer-related and general attitude-related constructs (see Table 1).

**Table 1:** Main questionnaire scales for dependent variables

Scale	Items	Factor 1 loading (attractiveness of the bank as an employer)	Factor 2 loading (general attitude toward the bank)
**Attractiveness of the bank as an employer **(adapted from Ageeva et al. (2018))	A job at this bank is very appealing to me	0.92	0.05
	For me, this bank would be a good place to work	0.90	0.07
	I would exert a great deal of effort to work for this bank	0.92	0.01
	This bank would be one of my first choices as an employer	0.95	− 0.02
	I would definitely accept a job offer from this bank if I were offered one	0.87	0.06
**General attitude toward the bank **(adapted from Brengman and Karimov (2012))	I believe that this bank is competent	0.05	0.91
	I believe that this bank understands the market it operates in	0.00	0.95
	I believe that this bank knows about banking products and services	0.02	0.95
	I believe that this bank knows how to provide excellent service	0.11	0.84

The subsequent confirmatory factor analysis validated this two-factor structure, demonstrating excellent fit indices (see Appendix C). Additionally, discriminant validity was confirmed by comparing the true-score correlations between factors to the square roots of the average variance extracted (AVE). The correlation between the constructs (*r* = 0.752) was below the square roots of AVE (0.943 for *general attitude toward the bank* and 0.935 for *attractiveness of the bank as an employer*), indicating acceptable discriminant validity.

Given the high reliability and validity of the scales, we calculated the mean scores for general attitude and attractiveness. These mean scores were then utilized in subsequent analyses to examine the relationships between webpage prototypicality, message strength, cognitive elaboration, and users’ attitudes toward the organization. Table 1 presents the questionnaire scales for the two main dependent variables, detailing each measurement item to ensure clarity and replicability, along with the factor loadings of the exploratory factor analysis.

To support the interpretation of our results, we adopted three items on persuasive impact by Pham and Avnet (2004, p. 514) to measure the *persuasiveness* of each of the three information elements provided: screenshots of the webpages, “About Us” statements, and employer reviews. We presented four small example screenshots for each element (e.g., “The “About Us” statements…”; anchored at “did not influence/influenced my opinion about the bank.”).

Additionally, we collected demographic data (i.e., *age*, *gender*, and *education*) and controlled for *familiarity*. When rating each bank, participants indicated their familiarity with that particular bank using a single seven-point Likert-scale item (not at all, very much) (Loken & Ward, 1990). Unlike Goodstein (1993), who included familiarity as a factor in advertisement evaluations, we controlled for familiarity by excluding highly familiar brands from our analysis. This approach was chosen because of the correlation between prototypicality and familiarity, as commonly encountered instances (high familiarity) tend to be viewed as prototypical.

### Participants

We recruited 107 participants via Prolific, a professional sample supplier. We restricted participation to people from the UK and the USA and aimed for an equal gender distribution. On average, each respondent took 23 min and 16 s to complete the study. Table 2 provides an overview of the participants’ descriptive characteristics by experimental condition. Among these 107 participants, 94% answered all six attention checks correctly, while the remaining six participants (6%) answered five of the six checks correctly. In the low elaboration condition, participants’ working memory performance indicates that they indeed paid attention to the concurrent task, as they correctly recalled an average of 80.82% of the dots (SD = 10.84%), with individual scores ranging from 61 to 98% (*N* = 51).

**Table 2:** Sample distribution

	Experimental condition		Total
	Low elaboration condition (with secondary working memory task)	High elaboration condition (without secondary working memory task)	
**Gender — Female**	51%	49%	
**Age (SD)**	41.55 (14.07)	38.50 (14.75)	
**Education**	No schooling (2%) High school graduate (20%) 1 or more years of college/university (26%) Bachelor’s degree (39%) Master’s degree (14%) Doctorate degree (0%)	No schooling (2%) High school graduate (23%) 1 or more years of college/university (23%) Bachelor’s degree (27%) Master’s degree (21%) Doctorate degree (4%)	
***N***	51	56	107

For the subsequent analysis, we discarded data related to banks with which participants reported high familiarity (i.e., rating over four on a seven-point scale, ranging from “not at all familiar” to “very much familiar”). This step was taken to prevent familiarity from distorting the results, reducing the dataset by 11%, from 1.712 to 1.529 data points. (We also conducted the analyses using only the data points where familiarity was rated as “not at all,” which yielded similar significance values.)

## Analysis and results

We used linear mixed models provided by the packages lme4 (Bates et al., 2020) and lmerTest (Kuznetsova et al., 2017) in R (www.r-project.org) to test the hypotheses on the non-aggregated data and analyze the influence of webpage prototypicality (high and low) and message strength (weak and strong) and their interaction on participants’ general attitude toward the bank and their perception of the bank as an attractive employer. In our models, we included prototypicality, message strength, and message type (“About Us,” employer review), which were effect-coded as − 0.5 and 0.5 to facilitate the interpretation of main effects and interactions. To control for the random position of webpages presented, we added the centered variable webpage position as a fixed effect. ParticipantID and webpageID were included as random effects.

To compare the effects of prototypicality under conditions of high and low elaboration, we divided participants into two experimental groups—one that completed a working memory task and one that did not—and performed separate analyses for each group. This approach simplified the interpretation of the results, as no three-way interaction effects had to be analyzed. Nevertheless, we additionally report the results of a linear mixed model analysis, taking all three independent variables into account at the same time (see Table 5).

Table 3 presents the analyses for the dependent variable employer attractiveness, while Table 4 shows the results for the dependent variable general attitude toward the bank. The *R*^2^ values for the analyses showed that between 43 and 51% of the variance in general attitude and employer attractiveness could be explained by the fixed and random effects.

**Table 3:** Results of linear mixed models analyses for attractiveness as an employer

	Attractiveness of the bank as an employer (low elaboration/with working memory task)			Attractiveness of the bank as an employer (high elaboration/without working memory task)		
Predictors	Estimates	CI	*p*	Estimates	CI	*p*
(Intercept)	4.33	4.01 to 4.65	** < 0.001**	4.36	4.05 to 4.67	** < 0.001**
High vs. low prototypicality	0.54	0.16 to 0.93	**0.006**	0.52	0.10 to 0.94	**0.015**
Strong vs. weak message strength	0.68	0.49 to 0.86	** < 0.001**	0.60	0.43 to 0.77	** < 0.001**
Prototypicality × message strength	− 0.37	− 0.75 to 0.01	0.057	0.02	− 0.33 to 0.36	0.932
*Control variable:* employer review vs. “About Us” message	0.09	− 0.10 to 0.27	0.349	0.34	0.17 to 0.51	** < 0.001**
*Control variable:* webpage position	− 0.03	− 0.05 to − 0.01	**0.009**	− 0.04	− 0.05 to − 0.02	** < 0.001**
Random effects						
*σ*^2^	1.59			1.42		
*τ*_00_	0.85 _ParticipantID_			0.77 _ParticipantID_		
	0.12 _WebpageID_			0.15 _WebpageID_		
ICC	0.38			0.39		
*N*	51 _ParticipantID_			56 _ParticipantID_		
	16 _WebpageID_			16 _WebpageID_		
Observations	738			791		
Marginal *R*^2^/conditional *R*^2^	0.082/0.430			0.078/0.442		

Values in bold indicate statistical significance at *p* < 0.05

**Table 4:** Results of linear mixed models analyses for general attitude toward the bank

	General attitude toward the bank (low elaboration/with working memory task)			General attitude toward the bank (high elaboration/without working memory task)		
Predictors	Estimates	CI	*p*	Estimates	CI	*p*
(Intercept)	5.04	4.71 to 5.36	** < 0.001**	5.00	4.68 to 5.31	** < 0.001**
High vs. low prototypicality	0.66	0.30 to 1.02	** < 0.001**	0.73	0.26 to 1.20	**0.002**
Strong vs. weak message strength	0.44	0.28 to 0.60	** < 0.001**	0.40	0.26 to 0.54	** < 0.001**
Prototypicality × message strength	− 0.38	− 0.72 to − 0.05	**0.024**	0.09	− 0.20 to 0.38	0.547
*Control variable:* employer review vs. “About Us” message	0.03	− 0.13 to 0.19	0.738	0.09	− 0.05 to 0.24	0.190
*Control variable:* webpage position	− 0.02	− 0.04 to − 0.00	**0.028**	− 0.03	− 0.05 to − 0.02	** < 0.001**
*σ*^2^	1.20			1.03		
*τ*_00_	0.95 _ParticipantID_			0.64 _ParticipantID_		
	0.11 _WebpageID_			0.21 _WebpageID_		
ICC	0.47			0.45		
*N*	51 _ParticipantID_			56 _ParticipantID_		
	16 _WebpageID_			16 _WebpageID_		
Observations	738			791		
Marginal *R*^2^/conditional *R*^2^	0.075/0.508			0.089/0.506		

Values in bold indicate statistical significance at *p* < 0.05

In both experimental conditions (high and low elaboration), there was a significant positive effect of prototypicality and message strength on user attitudes, supporting **H1** and **H2**. However, the associations tended to be slightly weaker in the high elaboration condition (i.e., without a working memory task). When users engaged in high elaboration, there was a main positive effect of webpage prototypicality (employer attractiveness: *b* = 0.52, *p* = 0.015; general attitude: *b* = 0.73, *p* = 0.002) and message strength (employer attractiveness: *b* = 0.60, *p* < 0.001; general attitude: *b* = 0.40, *p* < 0.001) on the attitudes toward the bank. In the low cognitive elaboration condition, prototypicality (employer attractiveness: *b* = 0.54, *p* = 0.006; general attitude: *b* = 0.66, *p* < 0.001) and message strength (employer attractiveness: *b* = 0.68, *p* < 0.001; general attitude: *b* = 0.44, *p* < 0.001) were also significant predictors.

**Fig. 3:**
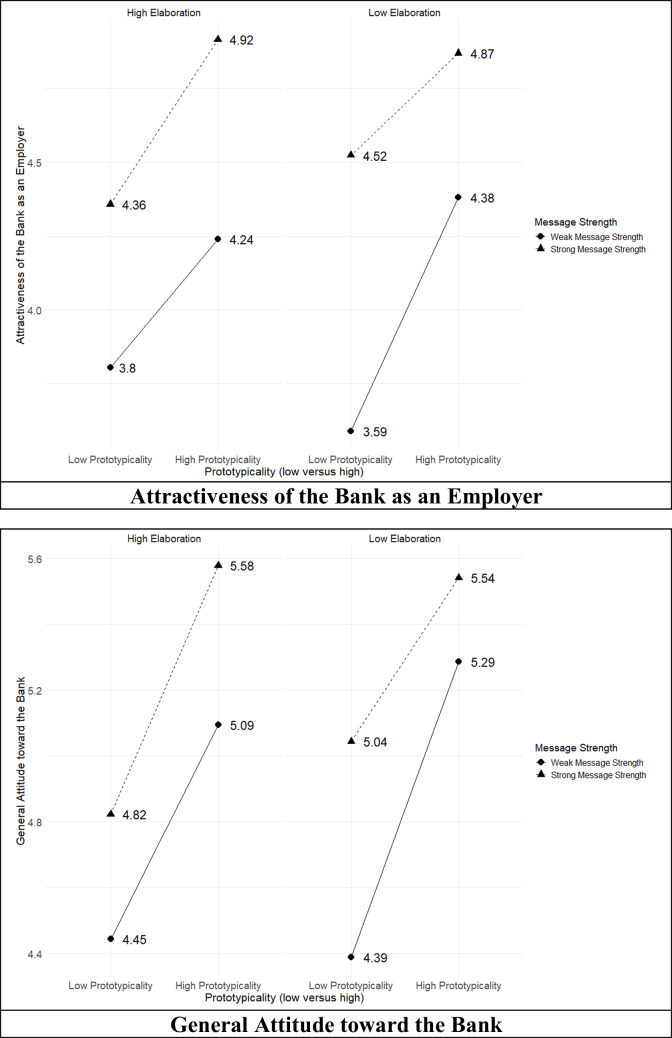
Interaction effect of prototypicality and message strength on the bank evaluation under different elaboration likelihood levels

Figure 3 illustrates these effects. Under high elaboration, when participants were not cognitively constrained, a strong message paired with high prototypicality led to the most favorable evaluation of the general attitude toward the bank and the attractiveness of the bank as an employer. Conversely, when prototypicality was low and the message was weak, the evaluation of the bank was lower. When the message was strong, the impact of prototypicality remained important, with high prototypicality resulting in a more favorable general attitude, compared to low prototypicality. These findings highlight that under high elaboration, both prototypicality and message strength significantly influence attitudes toward the bank, supporting **H1** and **H2**.

Since both factors were significant in both high and low cognitive elaboration conditions, we did not find support for **H3** and **H4**. Specifically, we did not observe that lower cognitive elaboration strengthened the effect of prototypicality (**H3**) or weakened the effect of message strength (**H4**).

The statistical models also tested **H5**, which predicted that low webpage prototypicality increases the effect of message strength on users’ attitudes toward organizations. Interestingly, in the low elaboration condition with the working memory task, there was an (almost) significant interaction effect between prototypicality and message strength (employer attractiveness: *b* = − 0.437,* p* = 0.057; general attitude:* b* = − 0.38,* p* = 0.024). Figure 3 shows how prototypicality reduced the effect of message strength on attitude, partially supporting **H5** in the case of low cognitive elaboration. That is, under low elaboration, prototypicality could buffer the effects of weak messages, but strong messages were still more effective.

The results supported **H6**, which hypothesized that cognitive elaboration would amplify the negative moderating effect of lower prototypicality on the impact of message strength on users’ attitudes toward organizations. When comparing the different analyses, we note that the interaction effect between prototypicality and message strength was more pronounced in the low elaboration condition than in the high elaboration condition, and it reached significance for the general attitude toward the bank. Our findings showed that when users were distracted and encountered a prototypical webpage, it did not matter as much for bank ratings whether the message was strong or weak. However, if the webpage looked unusual (low prototypicality), message strength made a considerable difference—strong messages had a substantial positive impact on bank ratings. Moreover, non-prototypical webpages resulted in lower average bank ratings compared to those with a prototypical design.

Figure 3 shows that in the low elaboration condition, the difference between strong and weak message strength was smaller when high prototypicality was present, compared to low prototypicality, where the difference was more pronounced. In the low elaboration condition, where participants were cognitively constrained (due to the working memory task), even with a weak message, high prototypicality led to a relatively favorable evaluation of the bank.

Next, we turn to the different types of messages on the webpage (employer reviews and “About Us” messages). As hypothesized in **H4**, the cognitive load experienced by users affected how these messages influenced their judgment of banks as potential employers. Interestingly, this influence was not observed for their general attitude toward banks (possibly because half of the messages were specific employer reviews and, therefore, might have a stronger influence on the perceived attitude of the bank as an employer). Under high elaboration, there was a significant interaction effect between the different message types (employer review, “About Us”) on the rating of employer attractiveness (*b* = 0.34,* p* < 0.001). The employer reviews had a stronger influence than the “About Us” messages. It should be noted that all employer reviews were positive (there were no negative ones), and they only varied in persuasiveness.

The control variable “position of the webpage”—reflecting the order in which participants rated 16 different webpages—also had a negative effect on user attitudes in both elaboration conditions. For employer attractiveness, webpages presented earlier in the sequence (i.e., those shown first) received more favorable ratings, while those presented later were evaluated more critically (low elaboration: *b* = − 0.03, *p* = 0.009; high elaboration: *b* = − 0.04, *p* < 0.001). A similar pattern was observed for general attitude toward the bank, where webpages presented later were rated more negatively (low elaboration: *b* = − 0.02, *p* = 0.028; high elaboration: *b* = − 0.03, *p* < 0.001). This suggests that participants became more critical as they progressed through the sequence of webpages, regardless of the elaboration condition.

However, the webpage position did not significantly alter the relationships between other variables, which were the focus of our hypotheses. The results of analyses with and without the webpage position variable were nearly identical, indicating that position did not confound the main effects. Although there was a significant position effect, this can largely be disregarded, as the order of presentation was randomly and evenly distributed across participants in both conditions. Therefore, any influence of presentation order is unlikely to have biased the results, supporting the robustness of the findings.

We also conducted analyses by adding the variable “Banks are generally a good place to work at” as an additional control variable. Positive attitudes toward banks positively influenced both the attractiveness of the bank as an employer and the general attitude toward the bank. However, the other results were not confounded, further demonstrating the robustness of our findings.

Table 5 presents the results of additional linear mixed models, incorporating cognitive elaboration (with/without working memory task) as an additional independent variable. Elaboration was effect-coded as − 0.5 and 0.5 to facilitate the interpretation of main effects and interactions. The results are consistent with previous analyses, so we focus on the description of the additional findings. There were no significant interaction effects between prototypicality and elaboration, nor between message strength and elaboration. However, the three-way interaction between prototypicality, working memory, and message strength was significant (*b* = − 0.44, *p* = 0.048) for the general attitude toward the bank. This indicates that the effect of prototypicality on general attitude toward the bank depends on both message strength and whether participants were in the high or low elaboration condition, providing clear support for **H6** regarding the dependent variable of general attitude toward the bank.

**Table 5:** Results of the linear mixed model analyses using elaboration as an additional factor

	Attractiveness of the bank as an employer			General attitude toward the bank		
Predictors	Estimates	CI	*p*	Estimates	CI	*p*
(Intercept)	4.35	4.08 to 4.61	** < 0.001**	5.02	4.75 to 5.28	** < 0.001**
High vs. low prototypicality	0.53	0.13 to 0.93	**0.009**	0.69	0.28 to 1.10	**0.001**
Low vs. high elaboration	− 0.03	− 0.39 to 0.34	0.893	0.04	− 0.31 to 0.40	0.805
Strong vs. weak message strength	0.64	0.52 to 0.76	** < 0.001**	0.42	0.31 to 0.53	** < 0.001**
High vs. low prototypicality × low vs. high elaboration	0.02	− 0.22 to 0.27	0.848	− 0.07	− 0.28 to 0.14	0.519
Strong vs. weak message strength × low vs. high elaboration	0.07	− 0.18 to 0.32	0.574	0.03	− 0.18 to 0.25	0.748
High vs. low prototypicality × strong vs. weak message	− 0.15	− 0.41 to 0.10	0.240	− 0.13	− 0.35 to 0.09	0.242
High vs. low prototypicality × strong vs. weak message × low vs. high elaboration	− 0.35	− 0.86 to 0.16	0.181	− 0.44	− 0.88 to − 0.00	**0.048**
*Control variable:* employer review vs. “About Us” message	0.22	0.09 to 0.34	**0.001**	0.07	− 0.04 to 0.17	0.206
*Control variable:* webpage position	− 0.03	− 0.04 to − 0.02	** < 0.001**	− 0.03	− 0.04 to − 0.01	** < 0.001**
Random effects						
*σ*^2^	1.50			1.10		
*τ*_00_	0.81 _ParticipantID_			0.79 _ParticipantID_		
	0.15 _WebpageID_			0.16 _WebpageID_		
ICC	0.39			0.46		
*N*	107 _ParticipantID_			107 _ParticipantID_		
	16 _WebpageID_			16 _WebpageID_		
Observations	1529			1529		
Marginal *R*^2^/conditional *R*^2^	0.078/0.438			0.0781/0.508		

Values in bold indicate statistical significance at *p* < 0.05

In the high elaboration condition (without the working memory task), low prototypicality combined with a weak message led to a sharp decline in attitudes. However, high prototypicality buffered against the negative impact of a weak message, resulting in a more positive attitude. In the high elaboration condition (without the working memory task), the reduced cognitive load improved participants’ ability to process these differences, resulting in less pronounced interaction effects between message strength and prototypicality. These results further lend support to **H6** in the case of general attitude toward the bank.

Table 6 presents participants’ perceptions of the influence of various study elements (screenshots, “About Us” messages, and employer review messages) under different elaboration likelihood conditions. On average, participants in the high elaboration group tended to perceive a slightly stronger influence of webpage elements (*M* = 5.74 for screenshots, *M* = 5.45 for “About Us” messages, and *M* = 5.67 for employer review messages) compared to the low elaboration group (*M* = 5.24 for screenshots, *M* = 4.96 for “About Us” messages and *M* = 5.32 for employer review messages). However, the differences in perceived influence between the two groups were not statistically significant, as indicated by the non-significant *t*-values, albeit *p*-values below 0.1 are often considered to suggest a trend (*t*_df=86.48_ = − 1.73, *p* = 0.088 for screenshots; *t*_df=76.41_ = − 1.75, *p* = 0.084 for “About Us” messages; *t*_df=89.47_ = − 1.32, *p* = 0.190 for employer review messages).

**Table 6:** Relevance of webpage elements (The table reports results for *t*-tests when equal variances are not assumed.)

Perceived influence	Elaboration likelihood	*N*	Mean	SD	*t*	df	*p*
Screenshots of webpages	Low (with working memory task)	51	5.24	1.75	− 1.73	86.48	0.088
	High (without working memory task)	56	5.74	1.18			
“About Us” messages	Low (with working memory task)	51	4.96	1.75	− 1.75	76.41	0.084
	High (without working memory task)	56	5.45	0.97			
Employer review messages	Low (with working memory task)	51	5.32	1.55	− 1.32	89.47	0.190
	High (without working memory task)	56	5.67	1.10			

These results help interpret the findings regarding **H3** and **H4**. Fundamentally, they support **H4**, as the relevance of the text parts was assessed as more influential under high cognitive elaboration. However, they essentially contradict **H4**, since the influence of the webpage screenshots was also assessed as more relevant under high cognitive elaboration. (It should be noted as a limitation here, that participants might not have been aware that the texts varied in message strength and the webpages in prototypicality when rating the influence of the different elements.)

## Discussion

This study investigated the collective influence of design variables through the concept of webpage prototypicality, rather than focusing on individual variables in isolation. Drawing on the ELM, we explored how webpage prototypicality interacts with message strength and users’ cognitive elaboration to affect attitude formation. Our results underscore the complex dynamics at play when users encounter webpages with varying degrees of prototypicality and message strength.

### Interpretation of results

We found that both in conditions of high and low cognitive elaboration, webpage prototypicality and message strength positively influenced users’ overall attitude toward the bank and how attractive the bank was perceived as an employer (supporting H1 and H2). Prototypical webpages consistently yielded positive effects, resulting in better bank ratings compared to non-prototypical webpages. They positively affected users’ impressions of banks as an attractive employer, independent of message strength. The positive effect of webpage prototypicality supports prior research that highlighted the important role of web design as a signal shaping user impressions (Ye et al., 2020) and attitudes (Ageeva et al., 2018) within digital environments.

While our initial hypotheses posited that elaboration likelihood would serve as a moderating factor for the effect of both prototypicality (H3) and message strength (H4) on users’ attitude toward organizations, our empirical findings did not support these assumptions. This stands in contrast to the predictions outlined in the ELM (Samson & Voyer, 2012). Notably, message strength, which demands more processing effort since text must be read, did not exhibit a significantly lower effect on user attitude as a central cue under low cognitive elaboration. Similarly, prototypicality, being an easily assessable peripheral cue, did not exert a stronger effect. A possible interpretation is that webpage prototypicality, rather than being perceived as a peripheral cue, might be considered as a central and important argument, which is strongly weighted even under high elaboration likelihood, exerting a positive and persistent impact on the central route of persuasion.

In addition, participants’ evaluations of the perceived influence of various webpage elements did not support H3. When they engaged in a concurrent working memory task (reducing their cognitive elaboration of webpages), users rated the perceived influence of webpage screenshots (differing in prototypicality) slightly lower. This observation could imply that their cognitive capacity to consciously include the webpages in their evaluations of banks was compromised. However, no direct conclusions can be drawn about the influence of prototypicality based on the participants’ evaluations of the perceived influence, as we only asked about the overall perceived influence at the end and not separately for each individual webpage. The effect was similar for participants’ evaluations of the influence of the messages. Here, the expected direction of the effect holds; users judged the influence of the messages as stronger under high elaboration likelihood.

Overall, the results suggest that under low elaboration likelihood, prototypicality plays a more important role in the evaluation of the bank as a good employer, while message strength becomes less important. Appendix B presents a summary of the supported and unsupported hypotheses.

Interestingly, when users’ cognitive elaboration was low, the impact of prototypical design took precedence over the message. This suggests that prototypicality is relied upon and can overshadow the influence of the message. In contrast, when the website had an atypical design, users paid closer attention to the message, making the strength of the message much more important. In this case, the difference between strong and weak messages was much clearer. On the other hand, when the design was typical, the difference between strong and weak messages was smaller. Overall, these results provide only partial support for H5 (specifically under conditions of low cognitive elaboration) and full support for H6.

In contrast, when the design was typical and the message was strong, users showed consistently positive attitudes toward the bank, underscoring the complementary role of both central and peripheral cues in shaping user perceptions. Our findings align with Goodstein’s (1993) work, which demonstrated that high prototypicality in ads led to less extensive cognitive processing as individuals relied on pre-existing schemas. Additionally, we incorporated message strength into the model, showing that strong textual content can influence user attitudes even when the website is atypical, suggesting that prototypicality and message strength can work together to shape perceptions, especially under low cognitive elaboration. Moreover, our study highlights that under low elaboration likelihood, users tend to rely more heavily on prototypicality as a cue, while message strength plays a more dominant role when the website is less prototypical. This finding is consistent with the idea that non-prototypical designs provoke suspicion or cognitive dissonance, leading to more careful consideration of central arguments (van Ooijen et al., 2016).

### Theoretical contributions and implications for research

This study offers several key theoretical contributions. First, the study’s conceptualization of prototypicality as a higher-level concept encompassing various webpage design variables extends the scope of prior research, which primarily focused on discrete webpage design variables as peripheral cues (Cyr et al., 2018; San José Cabezudo et al., 2009). This holistic approach enriches existing literature by providing a more comprehensive understanding of web design’s influence on attitudes.

Second, both prototypicality and message strength affected user attitudes, confirming the utility of the ELM in searching for potential predictors of attitude. Similar to the results of Schnurr et al. (2017), atypical design increased the relevance of message strength, which is consistent with one of four roles a design variable could play—affecting the choice of information processing type, in our case, skewing the choice toward the high-effort central processing. This observation further supports the application of the ELM, particularly to explore scenarios of high-distraction technology use, such as the use of mobile devices while on the move.

Third, prototypicality had a significant effect on user attitude under both low and high elaboration, implying that either prototypicality should have been considered as a central argument rather than a peripheral cue, or that peripheral and central processing are not mutually exclusive, but rather occur concurrently, supporting criticism of the ELM (Kitchen et al., 2014). While the ELM suggests that webpage prototypicality serves as a peripheral cue, secondary to textual arguments in the persuasion process (Kitchen et al., 2014), our study challenges this notion. Our results indicate that the impact of prototypicality can be comparable to that of persuasive arguments. In this respect, our results reinforce previous criticisms of the ELM, suggesting that message arguments and peripheral cues (Kitchen et al., 2014) have an influence together. It appears that the dynamics extend beyond a simple one-sided influence, where peripheral cues only influence the peripheral route, or message arguments exclusively influence the central route of persuasion. Notably, prototypicality demonstrated a dual role—as a standalone cue as well as a moderator influencing the elaboration of other arguments. This is in line with the assumptions of the ELM (Kitchen et al., 2014). However, clarifying the direction of the effect (i.e., which variable influences the other) was challenging based on the current study design, as we presented the message and the homepage simultaneously, and the participants could scroll back and forth. For instance, when participants read a weak message, they might have still been unsure about their assessment and based their evaluation on the webpage design. Conversely, if the message was strong and compelling, it did not make much difference whether the webpage was prototypical. Overall, the compounding negative effect of low prototypicality and weak message strength on user attitude toward an organization seems to be particularly pronounced under low elaboration likelihood.

Building on these observations, our study applied prototypicality within the ELM framework to generate several relevant insights for future research. Shifting from discrete webpage elements to an overarching concept of prototypicality may help researchers and practitioners more intuitively pinpoint why and how a design needs to be changed. Applying this broader conceptualization could also enrich related human–computer interaction (HCI) theories and models, such as those focused on user experience (Van Schaik & Ling, 2008), website first impression (Thielsch et al., 2014; Ye et al., 2020), persuasiveness (Kim & Fesenmaier, 2008), or the choice of digital products (Miniukovich & De Angeli, 2016). Future work could further validate the overarching concept of prototypicality across different digital interfaces, investigating its potential limitations in different design contexts. Furthermore, it could be valuable to assess its impact beyond attitudes, such as purchase intentions or user engagement.

### Implications for practice

This study provides valuable practical insights for businesses seeking to optimize their web design and effectively communicate their messages to potential employees. When deciding how to invest in web design, organizations essentially face two choices: (1) create a unique, potentially more expensive design, or (2) opt for a more standard, cost-effective approach using design templates or AI-based solutions. Aligning with our findings, when message strength is particularly high, a prototypical design can draw additional attention to the arguments under certain conditions, amplifying their impact without triggering counterarguments or skepticism, which might arise with a less prototypical design. Conversely, if the message is weak, maintaining a more prototypical design can help preserve a positive overall impression.

In the era of AI, web design is increasingly supported by generative AI technologies such as Sitekick’s AI and Durable, trained on extensive databases of high-converting landing pages (10Web, 2025; Durable, 2025; Sitekick, 2025). Similar to our process for identifying prototypical webpages (see Appendix A), these AI solutions replicate widely observed design patterns, thus producing highly prototypical pages. For industries like banking, where prototypicality can foster positive attitudes among potential employees, using AI-generated webpages could be advantageous. At the same time, organizations with exceptionally strong, persuasive messages might consider more customized designs if they wish to spotlight those arguments further. Ultimately, aligning both message strength and design approach, whether prototypical or more distinctive, helps ensure optimal user attitudes and engagement.

Moreover, the choice of message type warrants careful consideration when aiming to influence users’ perceptions of the company as an employer. For companies seeking to attract new employees, leveraging positive employer reviews alongside comprehensive “About Us” descriptions can be instrumental in shaping favorable perceptions among potential recruits.

### Limitations and future research

This study is subject to several limitations. First, the findings related to the general attitude toward the bank may be influenced by the unique characteristics of the banking services sector, which inherently involves higher risks for customers. These risks likely heighten users’ sensitivity to both webpage design and message content, potentially influencing information processing and attitude formation. This context-specific factor might limit the generalizability of our findings on general attitudes to other sectors with lower perceived risks; however, the generalizability of results related to the bank’s attractiveness as a potential employer should remain unaffected. Future research should explore whether similar dynamics can be observed in industries with different risk profiles, such as retail or entertainment. Nonetheless, the banking sector was particularly suitable for this study because banking products tend to be relatively uniform, thus reducing variability in customer preferences compared to industries like fashion. Moreover, in banking, prototypical designs are less likely to be perceived as boring, as customers tend to prioritize consistency and security over creativity. Another limitation arises from the separate presentation of the messages and the webpages, potentially signaling to participants that we expected these elements to influence their evaluations. An alternative approach might involve manually embedding the “About Us” messages into the webpages, while aiming to minimize distortions to the original visual design. However, this approach may not be applicable to employer reviews, as such reviews would typically not be displayed on a bank’s homepage. Furthermore, we did not obscure organization logos or remove references to organization names which would have minimized brand-related effects. We did this to keep the visual appearance as realistic as possible. To account for participant familiarity with the banks, we included familiarity as a control variable and discarded data related to banks with which participants reported high familiarity.

Regarding cognitive elaboration, although we were able to ensure low elaboration through a secondary task—which may still have been insufficiently demanding, given the limited support for our low-elaboration hypothesis—ensuring high elaboration proved more challenging, as it required a personal interest in working for a bank that may not have been present among all participants.

Using general-population participants instead of jobseekers with a background in finance could be a limitation, as jobseekers with financial experience may be more familiar with navigating banking-related webpages. Their greater exposure to such webpages might enable them to process the core message and disregard peripheral cues more efficiently. Lastly, using static webpage screenshots as stimuli may not fully capture the participants’ authentic experience of interacting with a real banking webpage. These static representations may not fully replicate the dynamic user experience of real-time navigation through a website.

Future research could delve deeper into the interplay between prototypicality and message strength, by exploring different environmental contexts or technological interfaces such as mobile websites.

## Conclusion

This research investigated the impact of webpage prototypicality, message strength, and elaboration likelihood on users’ attitude toward a prospective employer and the overall organization. Our findings from an online experiment (*N* = 107) underscore the positive impact of both high prototypicality and strong messages on users’ attitudes toward organizations. However, when users are mentally distracted, a prototypical webpage design remains influential, whereas the message becomes less important. In addition, our results suggest that under low elaboration likelihood, message strength assumes a greater role when the webpage is less prototypical. The presented findings provide insights for organizations to consider in their website design and communication strategies.

## Supplementary Information

Below is the link to the electronic supplementary material.

Supplementary file1 (DOCX 6.94 MB)

## Data Availability

The survey data supporting the findings of this study are available from the corresponding author upon reasonable request.

## Footnotes

1 https://aisnet.org/page/SeniorScholarListofPremierJournals
