# Supplementary material for: The danger of not being typical: The positive effect of webpage prototypicality on users’ attitudes
Source: Electron Mark. 2025 May 14;35(1):40. doi: 10.1007/s12525-025-00777-9 (PMC12078413; doi:10.1007/s12525-025-00777-9)
Supplement: Supplementary file 1 — Supplementary file1 (DOCX 6.94 MB) [file 12525_2025_777_MOESM1_ESM.docx]

Appendix

Contents

[Appendix A: Manipulation of Webpage Prototypicality 2](#_Toc196393461)

[Appendix B: Support for the Hypotheses 5](#_Toc196393462)

[Appendix C: Confirmatory Factor Analysis and Discriminant Validity 6](#_Toc196393463)

[Appendix D: Survey 8](#_Toc196393464)

[Appendix E: Experimental Materials 15](#_Toc196393465)

[Screenshots of All Bank Websites 15](#_Toc196393466)

[Eight High Prototypicality Websites 15](#_Toc196393467)

[Eight Low Prototypicality Websites 23](#_Toc196393468)

[Messages 31](#_Toc196393469)

[Eight Employer Reviews 31](#_Toc196393470)

[Eight About Us Statements 32](#_Toc196393471)

[Screenshots of All Workload Tasks 33](#_Toc196393472)

[References 37](#_Toc196393473)

# Appendix A: Manipulation of Webpage Prototypicality

To examine the role of a webpage’s prototypicality, we collected screenshots of webpages that varied in their level of prototypicality. The process involved several steps to ensure that the selected webpages effectively represented high and low prototypicality while controlling for potential confounding factors.

**Step 1: Collection of Webpage Dataset.** We acquired banking website URLs through multiple strategies, including search engine queries (e.g., ‘commercial bank in Australia’), extraction of URLs from Wikipedia’s banking company lists in English-speaking countries, and filtering of existing curated lists (e.g., list of all financial institutions in the United States). From each website, we sampled a single webpage—typically the homepage, known for its representativeness of the website’s overall design. We deliberately excluded landing pages like those directing users to country-specific websites or announcing that a website moved to another URL. Screenshots and multiple web design-related metrics were collected for each homepage. To facilitate this process, we developed a custom add-on for the Mozilla Firefox browser, which ‘cleaned’ a webpage (removing overlays, pop-ups, notifications, and other non-essential web elements), modified it to look natural as an image (e.g., removing or moving down ‘sticky’ bottom-of-the-screen elements or menus), and saved a full-length screenshot of it. In total, we collected a dataset of 1.032 bank webpages as a first step in selecting suitable stimuli for our study.

**Step 2: Crowdworker Evaluations of Homepages.** To select suitable webpages for our empirical study, we relied on evaluations from crowdworkers on MTurk. Traditional prototypicality measures often include classification time and proportion of misclassifications since higher-prototypicality items are easier and faster to classify as members of a category (San José Cabezudo et al., 2009). However, we opted for questionnaire-based assessments, as recording categorization times online could lead to noisy data, since crowdworkers might be distracted by various things when participating from home. Inspired by Loken and Ward (1990), prototypicality was measured with three items: typicality (i.e., how typical an item is for its category), exemplar goodness (i.e., how well an item is suitable as an exemplar for a category), and family resemblance (i.e., measuring the similarity in attributes an item shares with other members of its category).

We also measured various control variables, such as visual complexity, aesthetics, novelty, craftsmanship, and trustworthiness—factors known to affect webpage appreciation (e.g., Miniukovich & Marchese, 2020). These controls served two purposes: to account for potential confounding effects during subsequent webpage selection and to sub-select webpages of varying prototypicality levels (i.e., low vs. high) that shared similar complexity and aesthetics. These control variables were also used to filter out unusual webpages, e.g., those looking outdated, broken, or overly complex, thus minimizing the effects of potential confounding factors. Table 1 provides an overview of the evaluation dimensions for homepage screenshots.

| Scale | Item | Scale (from -3 to +3) | |
| --- | --- | --- | --- |
| Prototypicality 1: Typical Design | This webpage looks like a typical commercial-bank homepage. | Not at all | Very much |
| Prototypicality 2: Exemplar Goodness | This webpage is a representative example of commercial-bank homepages. | Not at all | Very much |
| Prototypicality 3: Family Resemblance | This webpage has many visual aspects in common with homepages of other commercial-bank websites. | Not at all | Very much |
| Visual Complexity | This webpage looks | Simple | Complex |
| Aesthetics | This webpage looks | Ugly | Beautiful |
| Novelty | This webpage looks | Outdated | Modern |
| Craftsmanship | This webpage looks | Amateurish | Professional |
| Trustworthiness | This webpage looks | Not trustworthy | Trustworthy |

Table 1: Evaluation dimensions for homepage screenshots

Since the motivation of crowdworkers to participate honestly is sometimes low, which can lead to low-quality or fraudulent data (Chmielewski & Kucker, 2020; Kennedy et al., 2020), we implemented several quality control measures. These measures encompassed gradual webpage scrolling (to ensure participants saw the entirety of a webpage before rating), checking user ratings for consistency (some webpages were rated twice and should have received a similar rating from the same participant both times if they paid attention to the task), checking if a participant could differentiate seen from unseen webpages after the main study, reviewing the free-form feedback left by participants, reviewing participants’ demographic data, and checking if a participant used a VPN or had an IP from a country that did not speak English.

Overall, 1.298 crowdworkers were recruited via the Amazon Mechanical Turk crowdsourcing platform, and each crowdworker evaluated 71 distinct webpages (the first three were for training and were identical for all participants). The dataset of crowdworker evaluations is available as an open dataset (citation blinded for review). To increase data quality, participants were tasked with evaluating only one dimension/item (e.g., only aesthetics or exemplar goodness) to minimize the need to mentally switch between dimensions and thus decrease cognitive load. This approach also shortened the duration of the experimental sessions. On average, each session lasted 20 minutes, with crowdworkers receiving 2.1 USD upon verification of data quality. We collected, on average, 4.5 ratings per stimulus per dimension for non-prototypicality items (e.g., aesthetics and visual complexity) and 6.5-7 ratings for the three prototypicality items (typicality, exemplar goodness, and family resemblance). Overall prototypicality was computed as the mean of these three dimensions.

**Step 3: Final Selection of Homepage Screenshots.** From the pool of 1.032 bank webpages, we identified two distinct groups of webpages representing high and low prototypicality. Within each group, we selected eight homepage screenshots. Our selection process was designed to minimize the correlations between prototypicality and potential confounding factors. For this purpose, we first fitted a linear model of prototypicality, with confounders as predictors (i.e., aesthetics, complexity, craftmanship, and novelty), then used the model’s residuals as a confounder-independent prototypicality. 50 webpages from both extreme ends of the resulting confounder-independent prototypicality distribution were sub-sampled in the high/low prototypicality groups, which were then further reduced to eight per group using several selection criteria. The inclusion criteria for the final set of stimuli ensured that only less familiar banks (with less than 30 branches) were included, thereby avoiding well-known large international banks. Additionally, the number of bank branches was balanced between groups (high/low prototypicality). Furthermore, we excluded banks that were not exclusively commercial (e.g., those also offering investment and wealth management, or insurance services) and retained only US-based banks, as the country could be an additional confounding factor. Figure 1 presents examples of bank webpages for each group.


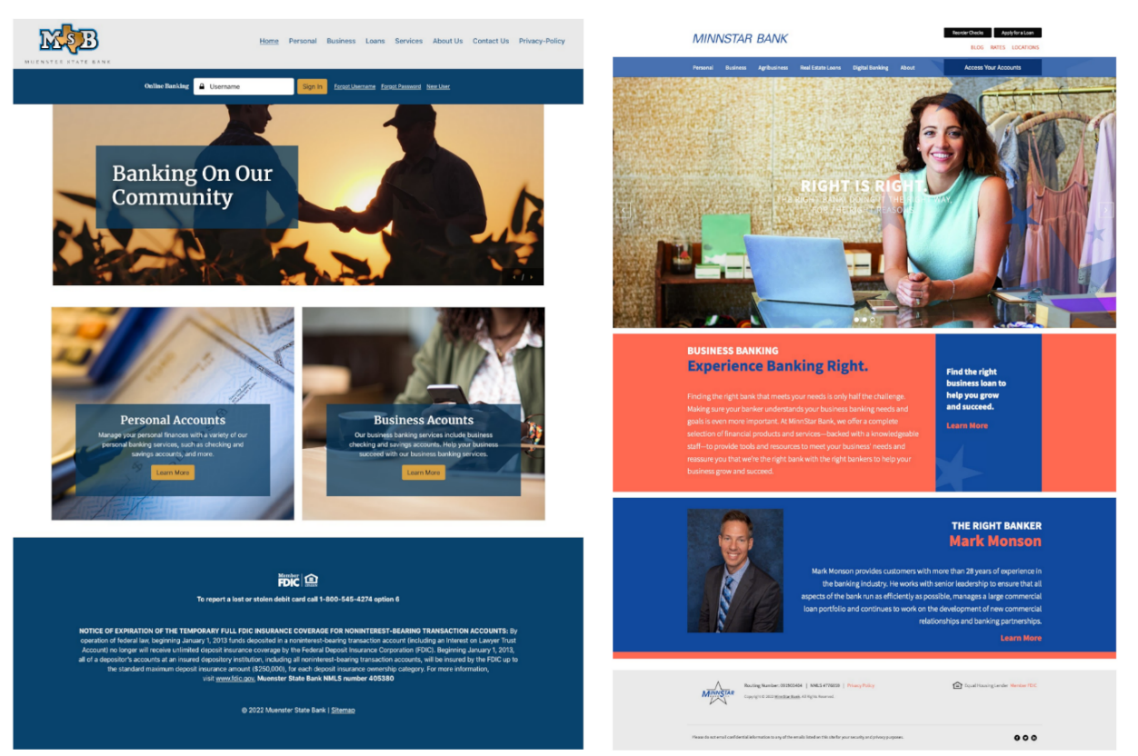


Figure 1: Example screenshots for a high prototypical webpage (left) and a low prototypical webpage (right) of a bank

# Appendix B: Support for the Hypotheses

| Hypotheses | Support |
| --- | --- |
| H1: Webpage prototypicality positively affects users’ attitudes toward an organization. | Supported. |
| H2: Message strength positively affects users’ attitudes toward an organization. | Supported. |
| H3: Lower cognitive elaboration strengthens the effect of webpage prototypicality on users’ attitudes toward an organization. | Not supported. |
| H4: Lower cognitive elaboration weakens the effect of message strength on users’ attitudes toward an organization. | Not supported. (However, partially supported by users’ evaluations of the perceived influence of ‘About us’ messages.) |
| H5: Lower prototypicality of a webpage increases the effect of message strength on users’ attitudes toward an organization. | Supported only under low cognitive elaboration. |
| H6: Lower cognitive elaboration amplifies the negative moderating effect of lower prototypicality on the effect of message strength on users’ attitudes toward an organization. | Supported for the general attitude toward the bank. |

Table 2: Support for the hypotheses

# Appendix C: Confirmatory Factor Analysis and Discriminant Validity

A confirmatory factor analysis (CFA) was performed to assess the two-factor structure from the exploratory factor analysis. The model included **general attitude toward the bank** (4 items) and **attractiveness of the bank as an employer** (5 items). Fit indices indicated excellent model fit: Comparative Fit Index (CFI) = 0.989, Tucker-Lewis Index (TLI) = 0.984, Root Mean Square Error of Approximation (RMSEA) = 0.076 (90% CI: 0.067–0.084), and Standardized Root Mean Square Residual (SRMR) = 0.017. Although a chi-square test was significant (χ²(26) = 253.657, p < 0.001), this was expected given the large sample size (N = 1529). All items loaded strongly and significantly on their respective factors, with standardized loadings ranging from 0.927 to 0.957 for general attitude toward the bank and 0.919 to 0.960 for attractiveness of the bank as an employer (see Table 3).

| Scale | Items | Unstandardized loading | Standardized loading |
| --- | --- | --- | --- |
| Attractiveness of the bank as an employer (adapted from Ageeva et al. (2018)) | A job at this bank is very appealing to me. | 1.624 | 0.960 |
|  | For me, this bank would be a good place to work. | 1.566 | 0.951 |
|  | I would exert a great deal of effort to work for this bank. | 1.623 | 0.922 |
|  | This bank would be one of my first choices as an employer. | 1.622 | 0.929 |
|  | I would definitely accept a job offer from this bank if I were offered one. | 1.590 | 0.919 |
| General attitude toward the bank (adapted from Brengman and Karimov (2012)) | I believe that this bank is competent. | 1.426 | 0.944 |
|  | I believe that this bank understands the market it operates in. | 1.495 | 0.943 |
|  | I believe that this bank knows about banking products and services. | 1.493 | 0.957 |
|  | I believe that this bank knows how to provide excellent service. | 1.454 | 0.927 |

Table 3. CFA factor loadings

An alternative CFA model incorporating a common method factor was tested but encountered significant identification issues, including a non-positive definite variance-covariance matrix and negative variances for some items. These problems suggest over-specification, likely due to the high inter-factor correlation. Consequently, the original two-factor model was retained.

Both constructs demonstrated high reliability, with Cronbach’s alpha values of 0.969 (general attitude toward the bank) and 0.972 (**attractiveness of the bank as an employer**). The average variance extracted (AVE) values were 0.888 and 0.875, respectively.

The covariance between general attitude toward the bank and attractiveness of the bank as an employer was substantial (Covariance = 0.752), indicating a strong positive relationship between the two constructs. Discriminant validity was confirmed as the square roots of AVE (0.943 and 0.935) exceeded the inter-construct correlation (r = 0.752), indicating that the constructs are distinct (see Table 4).

|  | General attitude toward the bank | Attractiveness of the bank as an employer |
| --- | --- | --- |
| **General attitude toward the bank** | 0.943 | 0.752 |
| **Attractiveness of the bank as an employer** | 0.752 | 0.935 |

Table 4. Discriminant validity assessment

# Appendix D: Survey


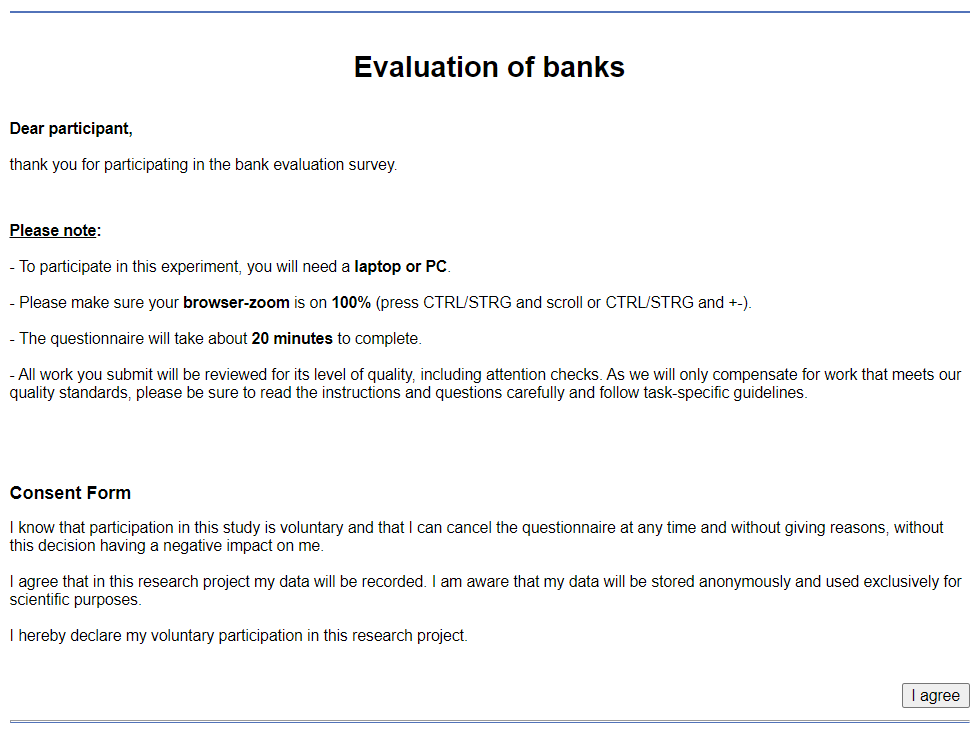


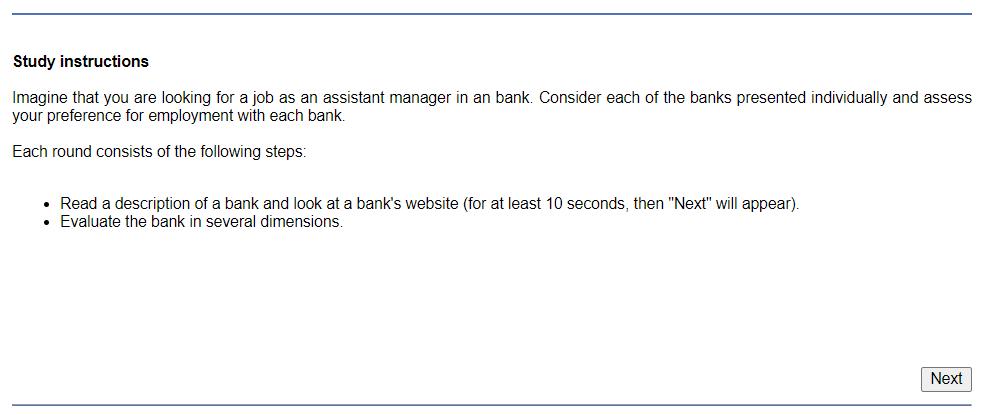


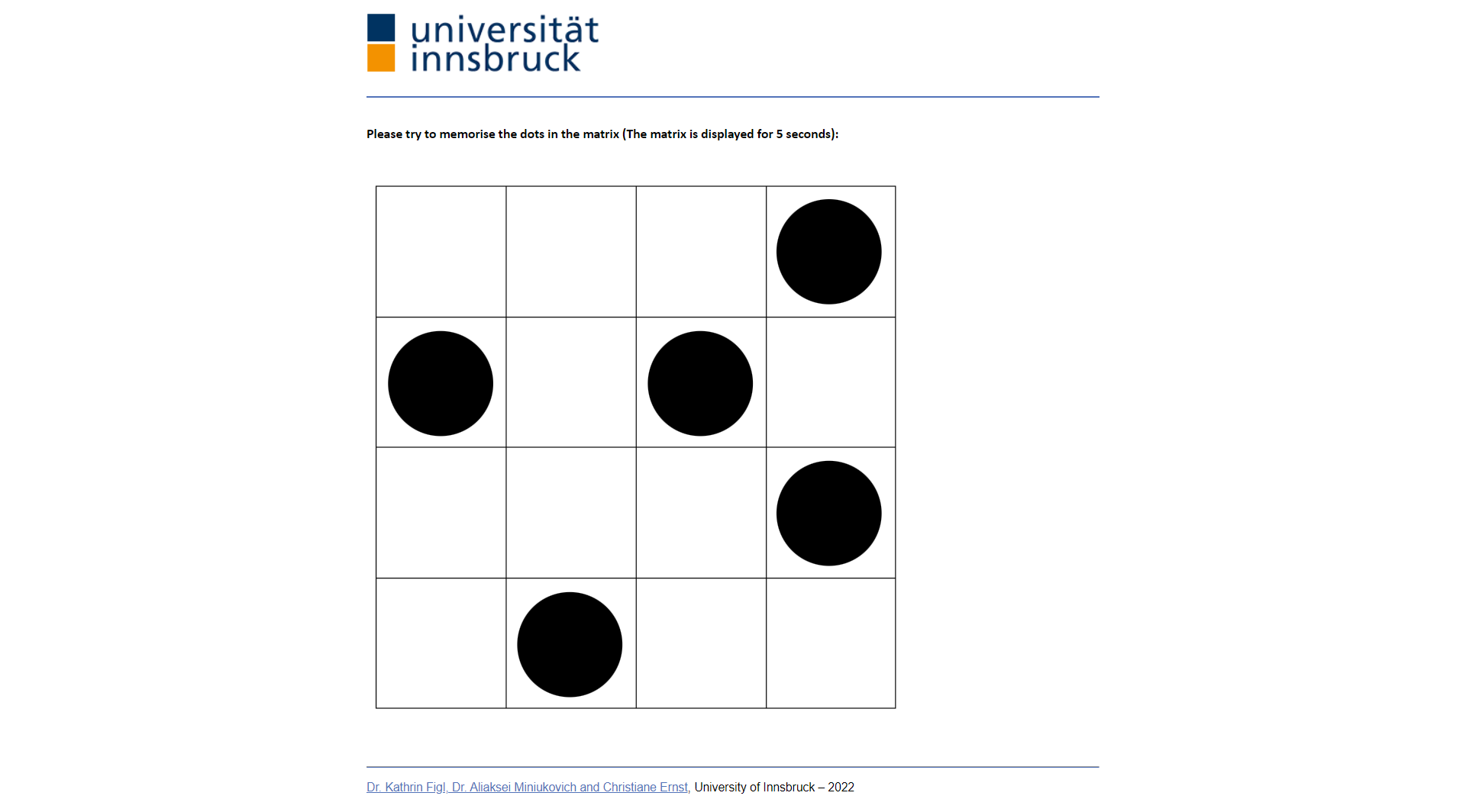

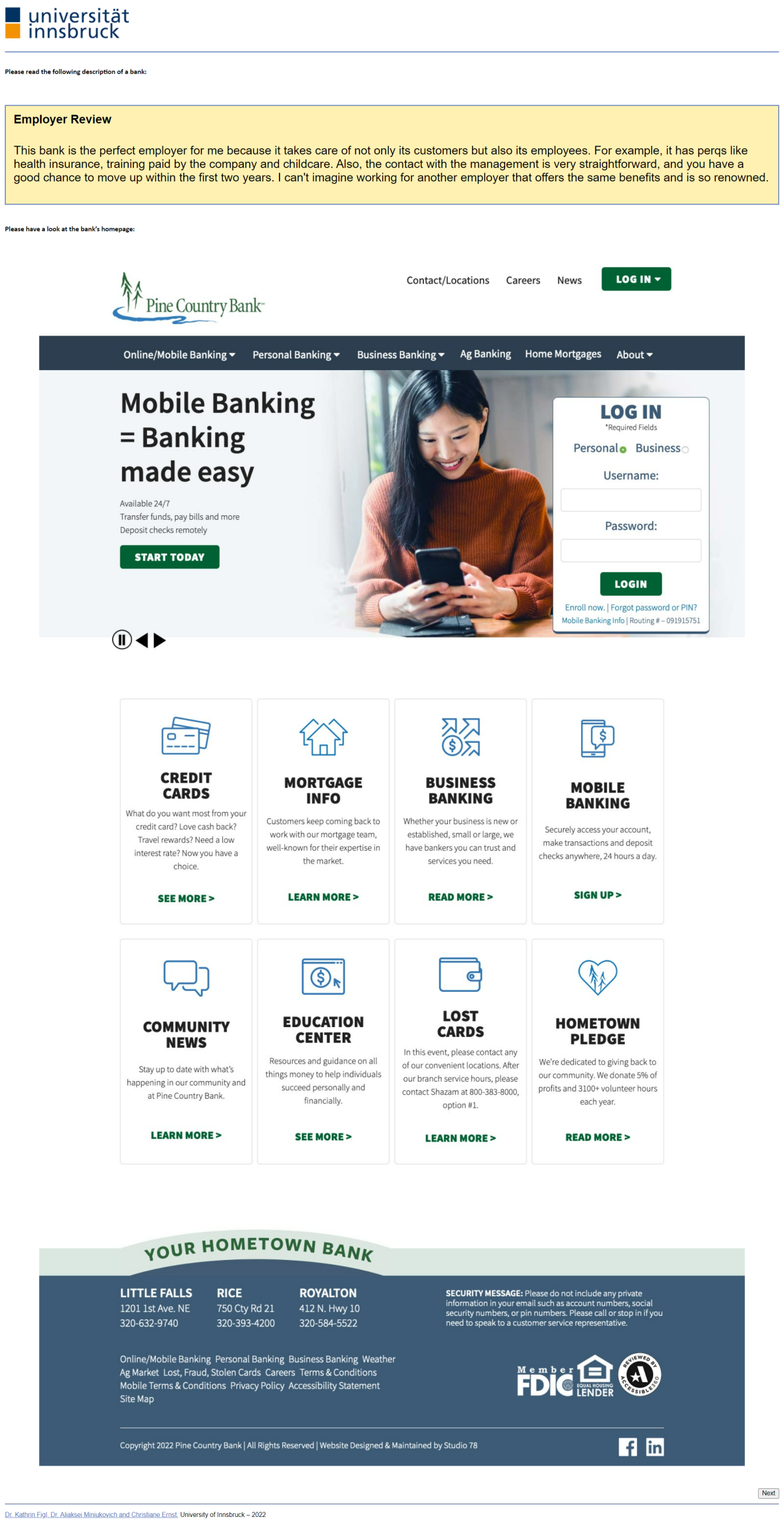


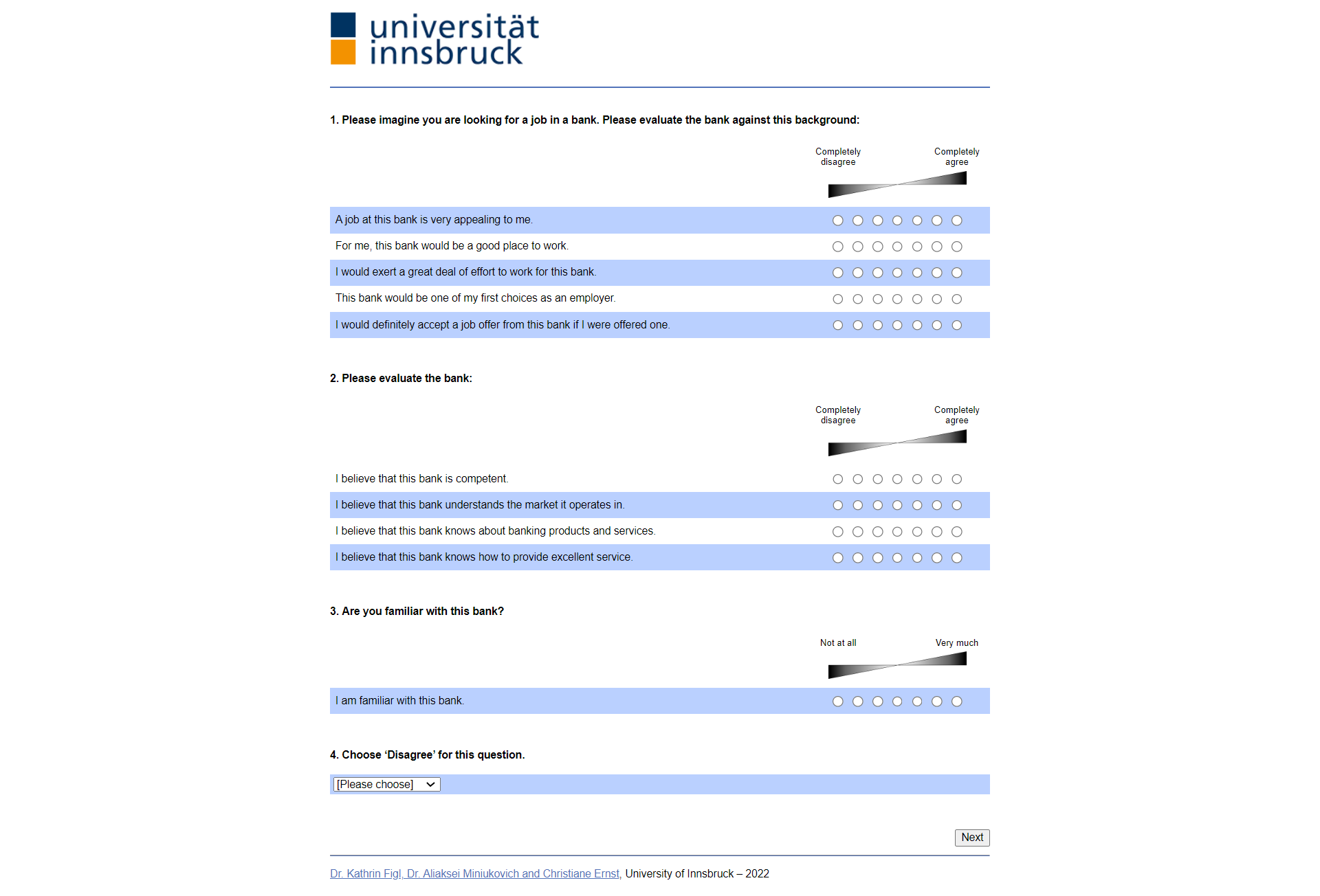


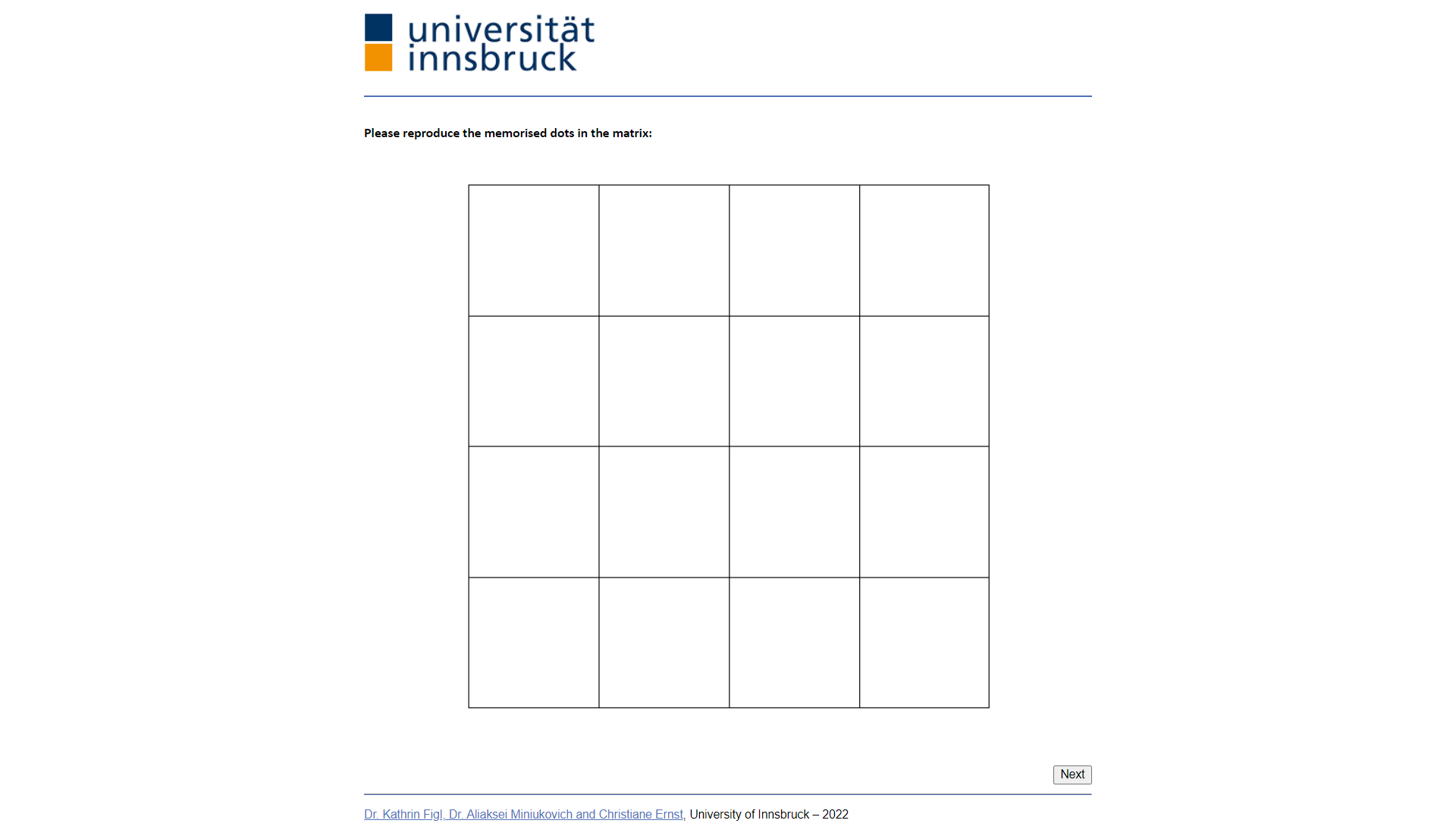


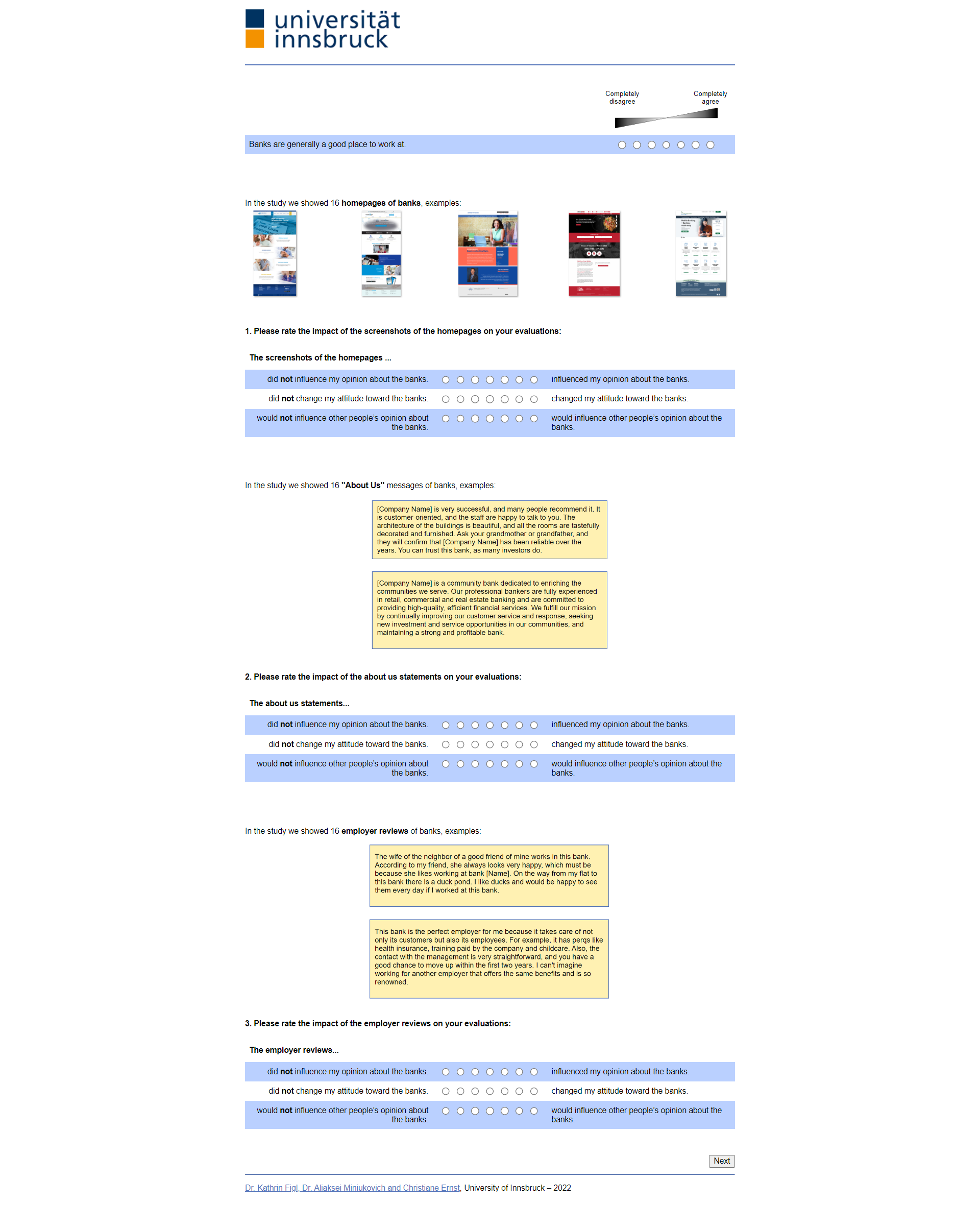


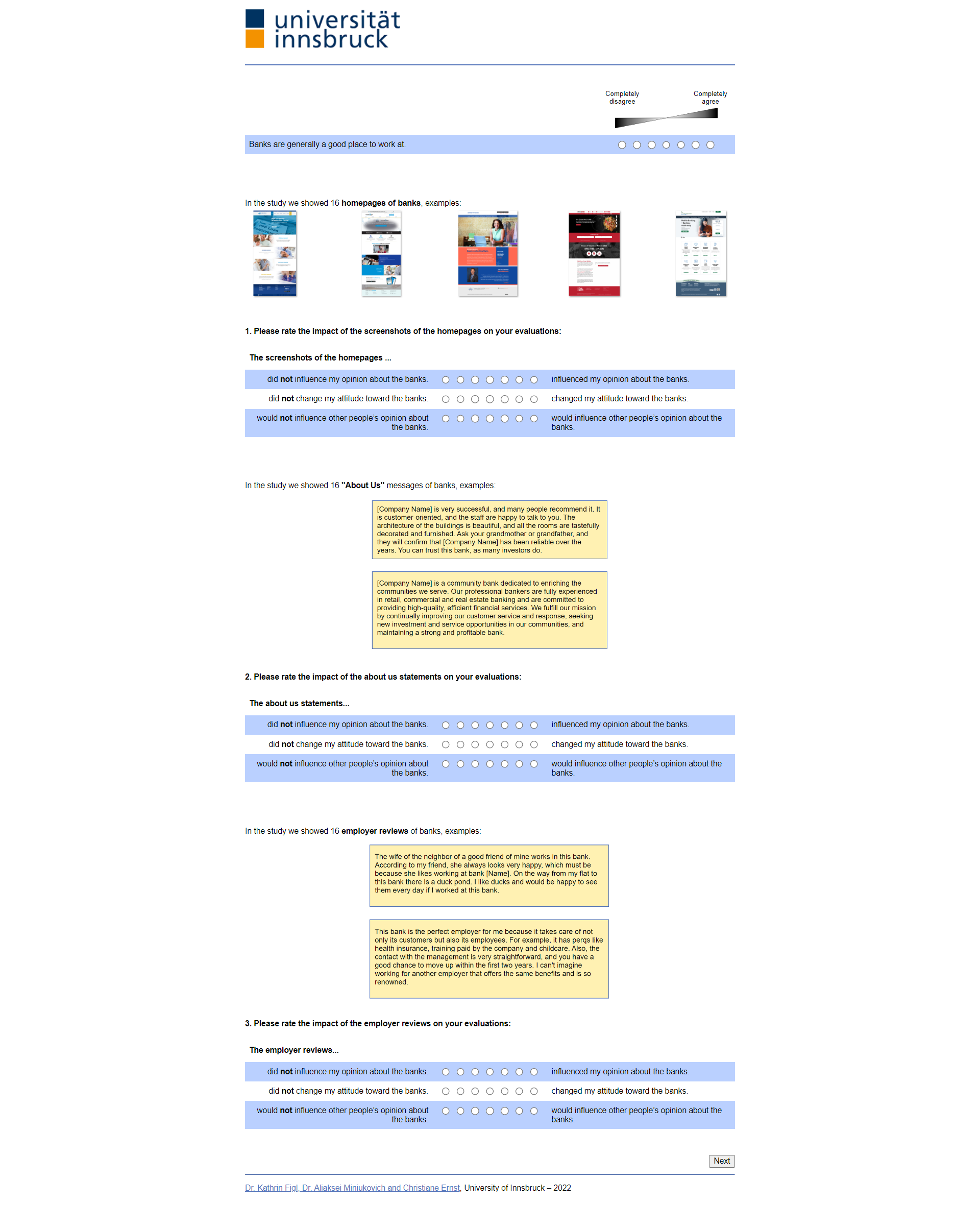


# Appendix E: Experimental Materials

## Screenshots of All Bank Websites

### Eight High Prototypicality Websites


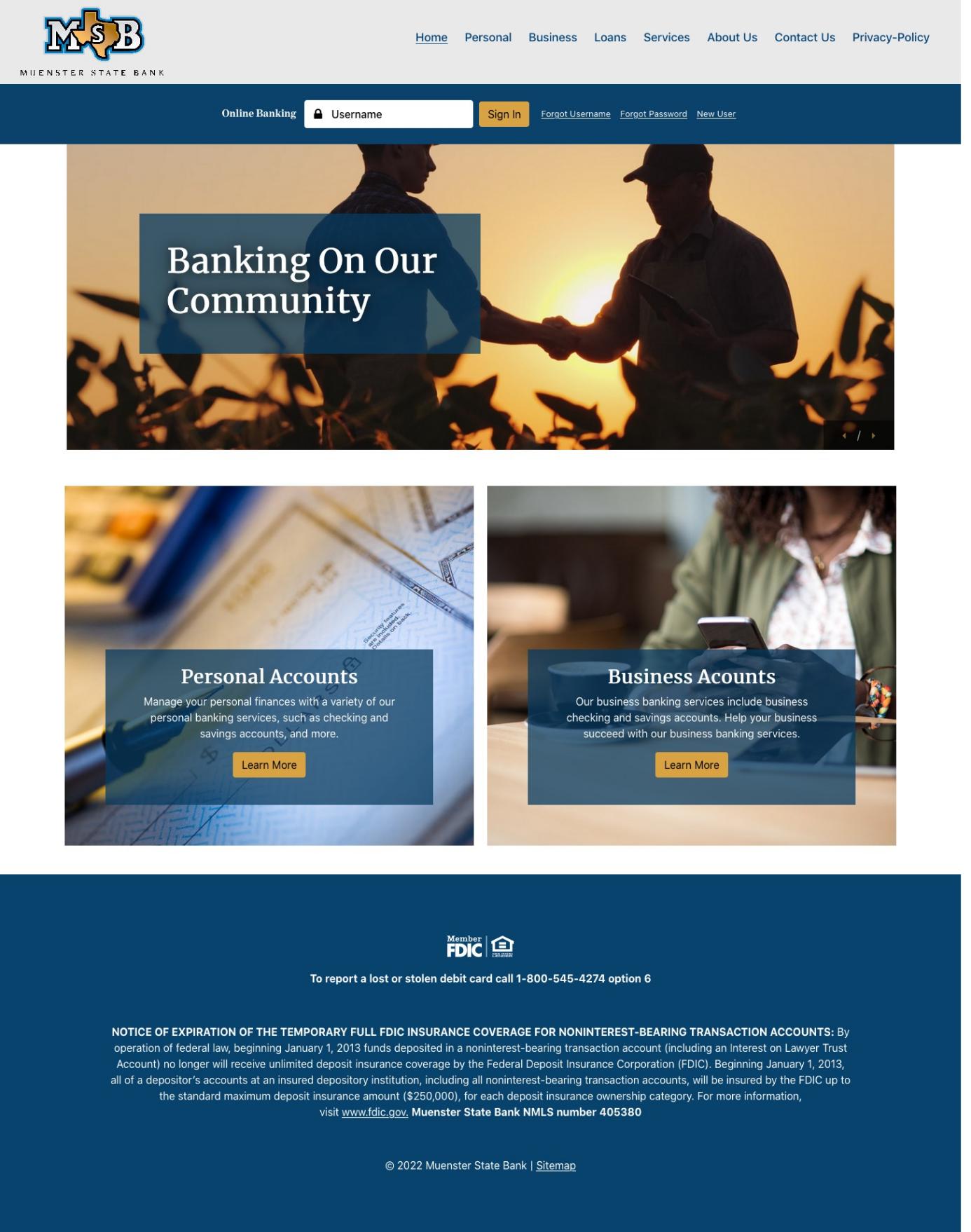


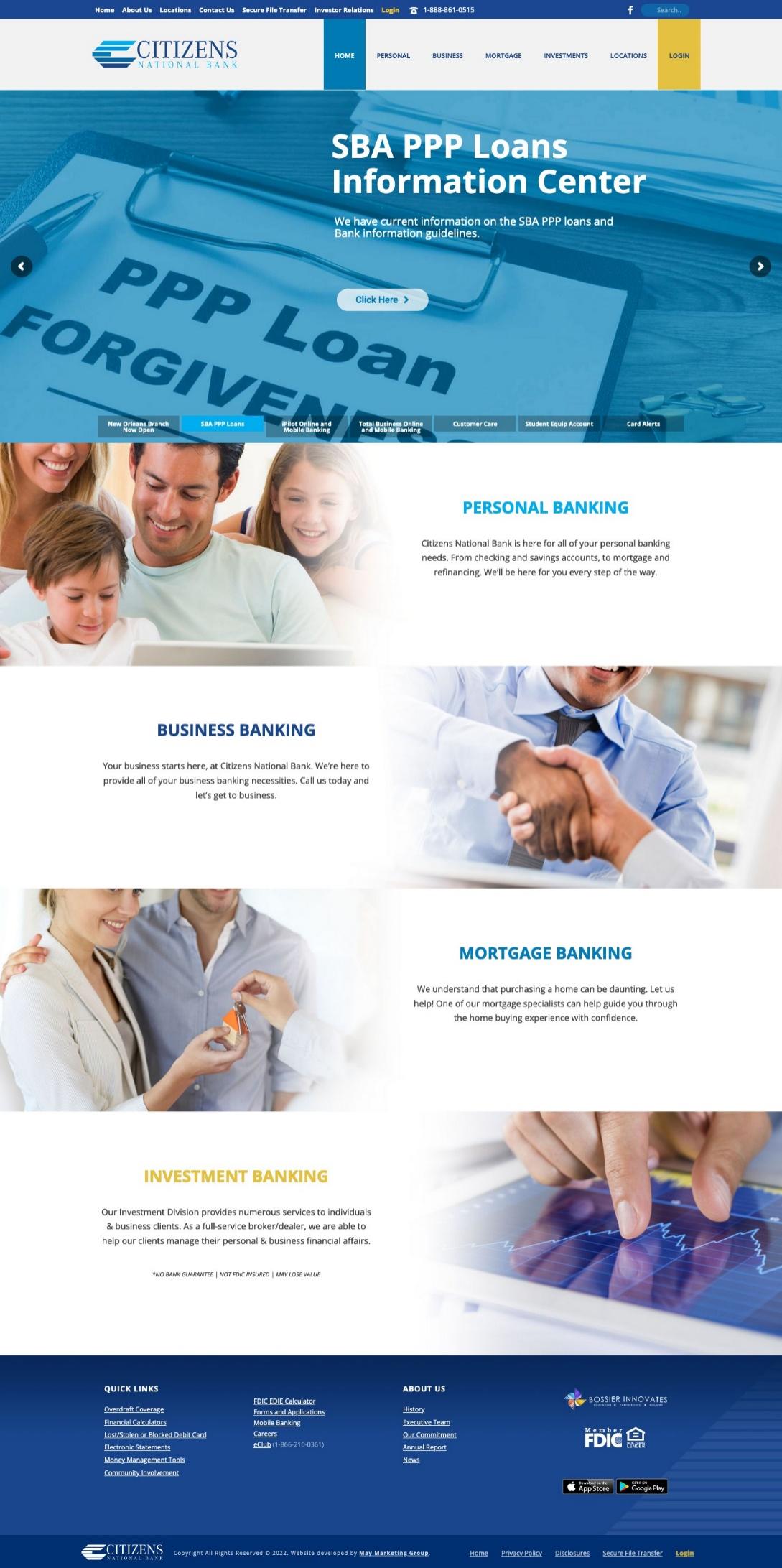

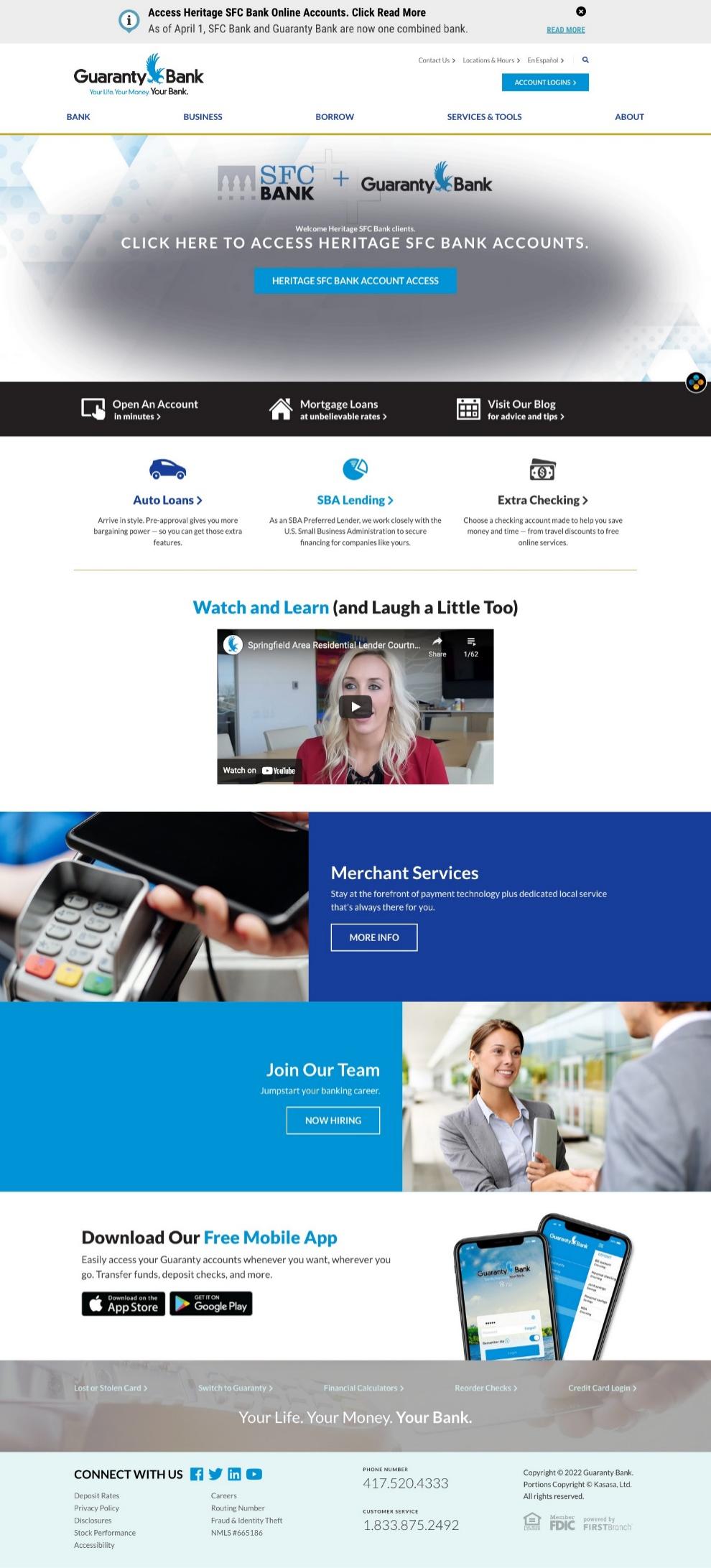


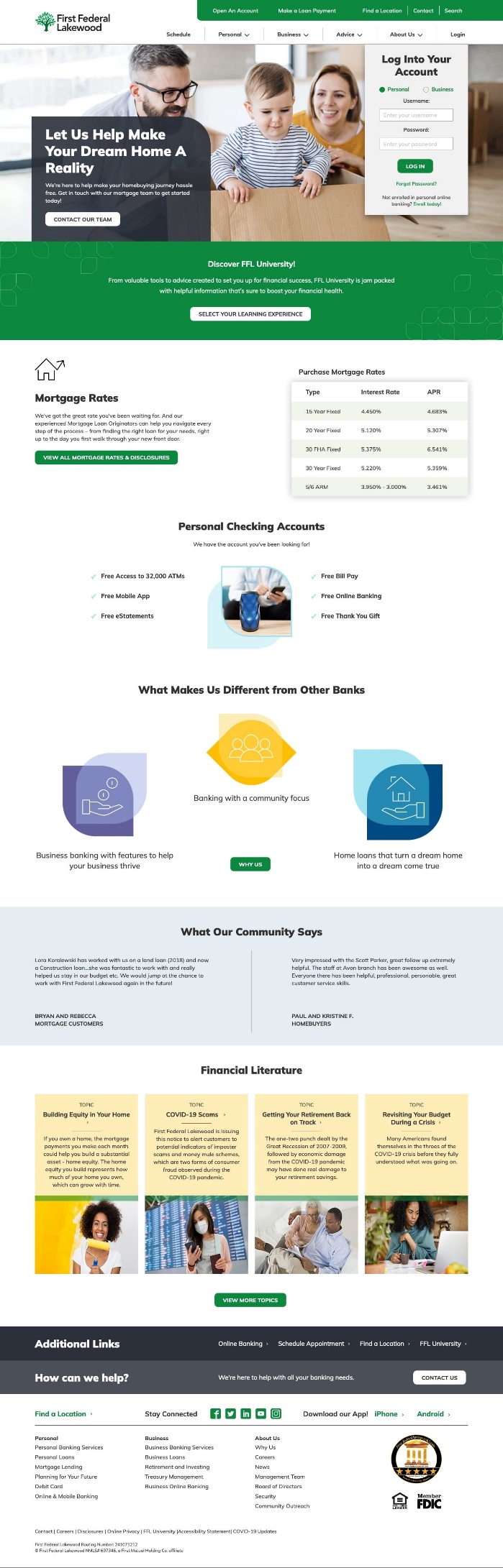


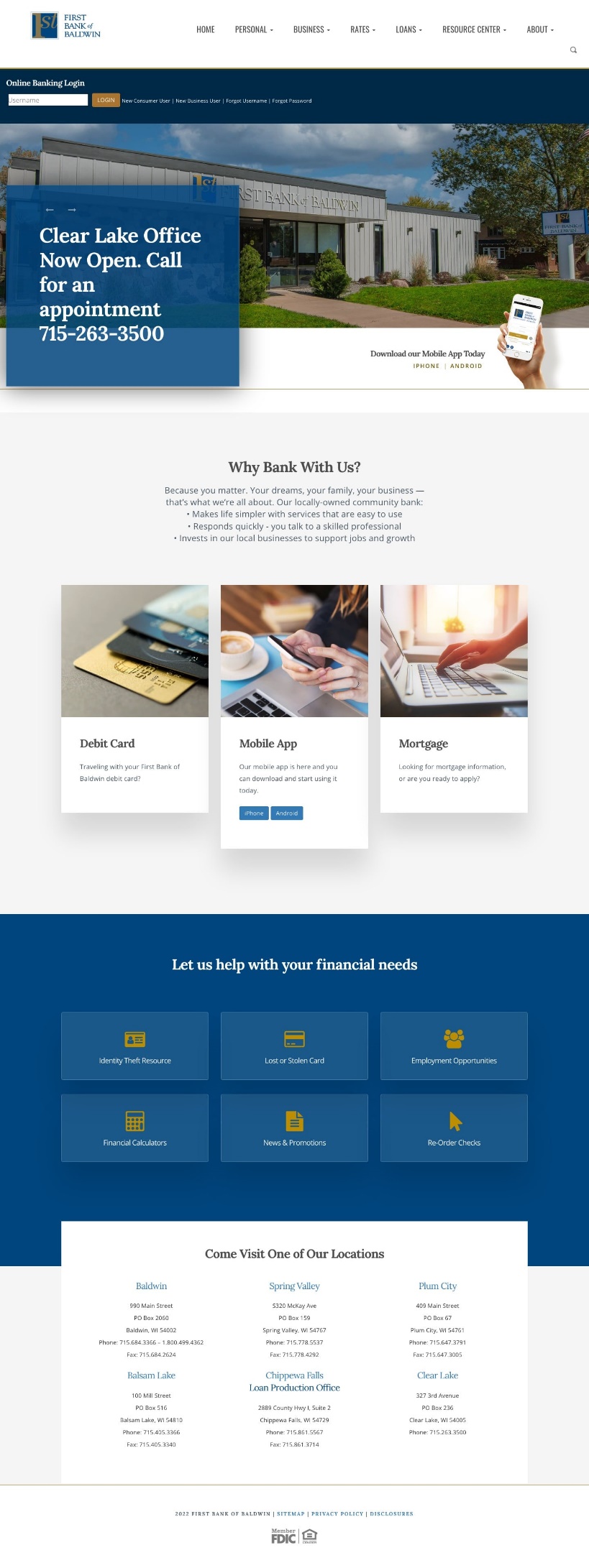


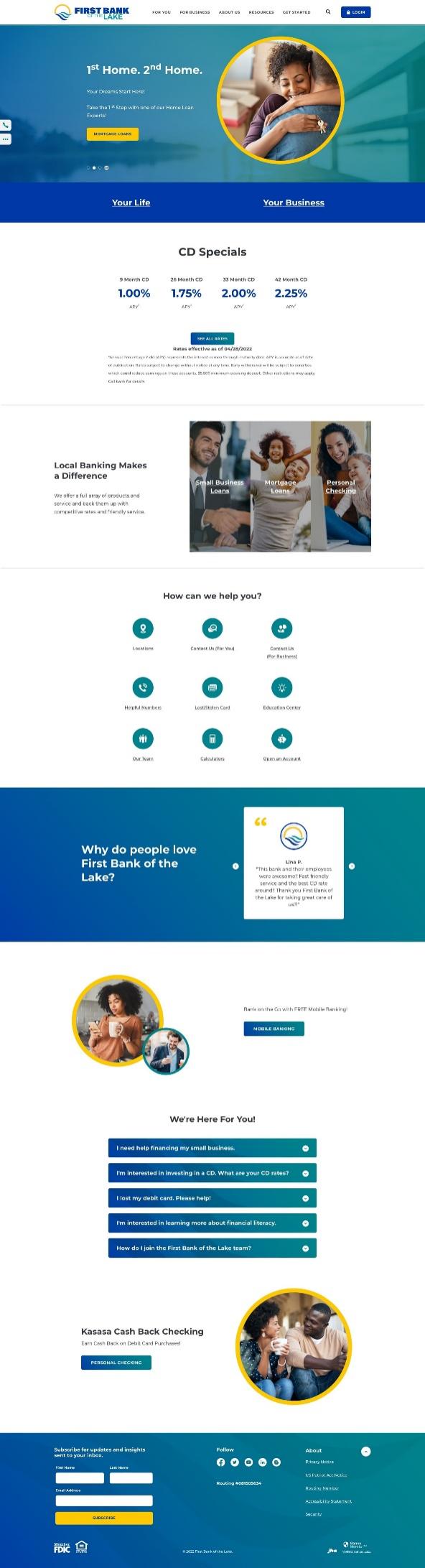


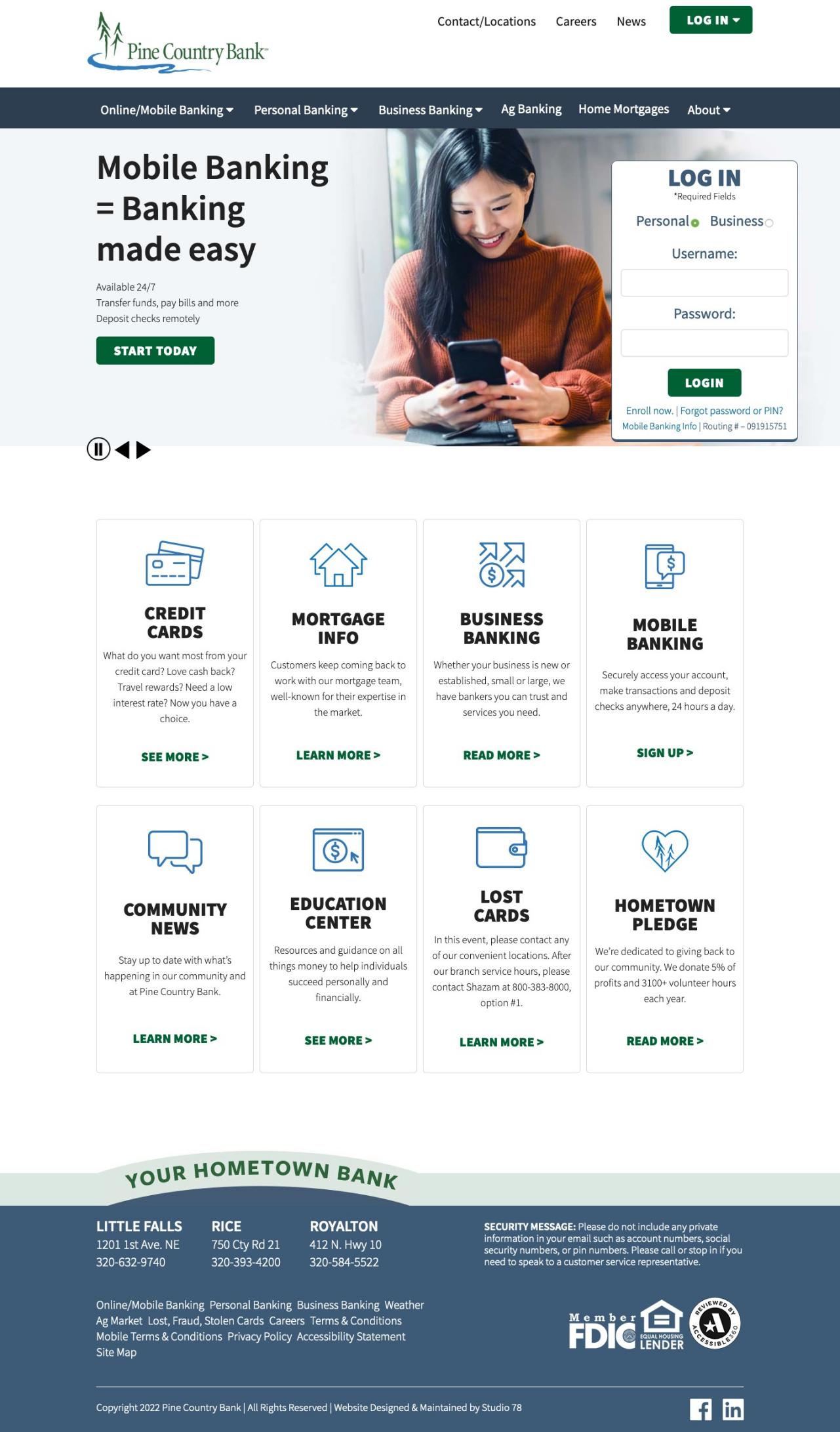


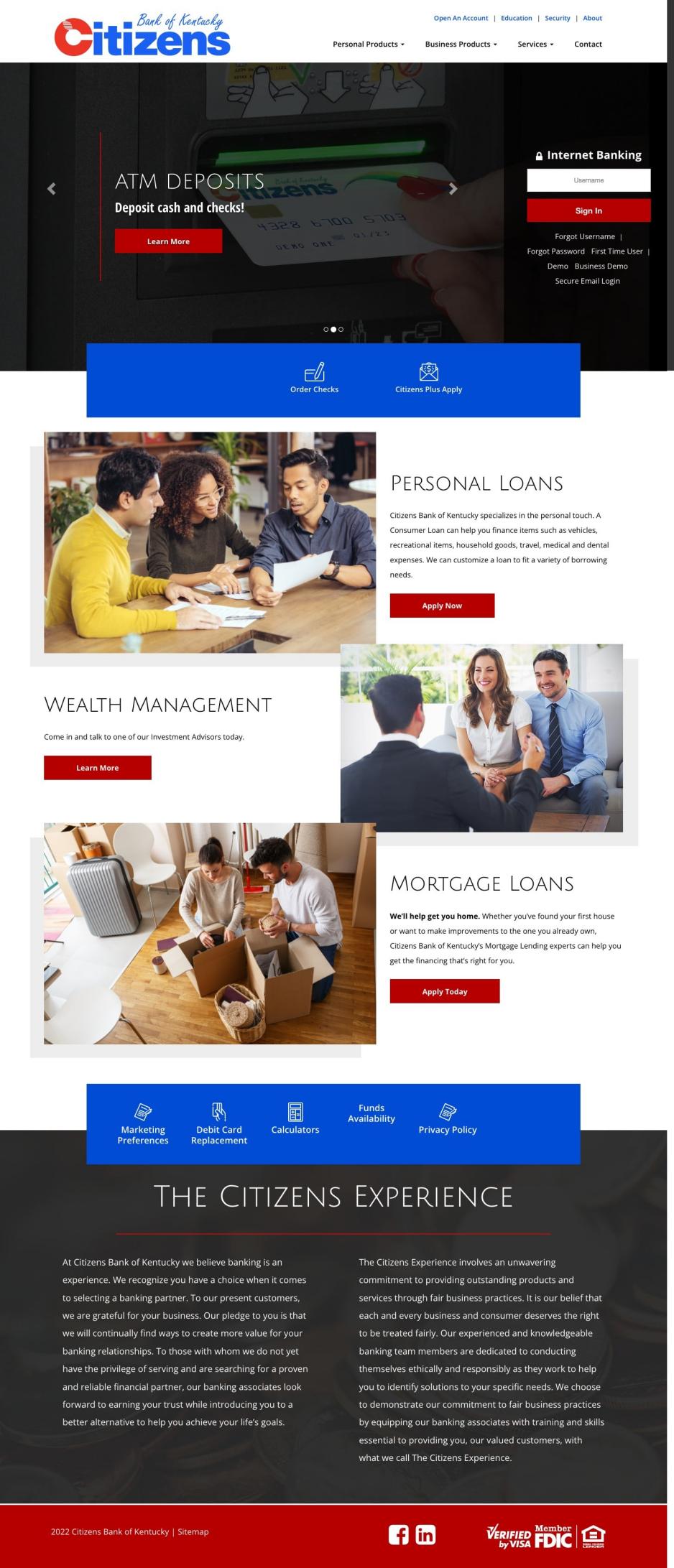


### Eight Low Prototypicality Websites


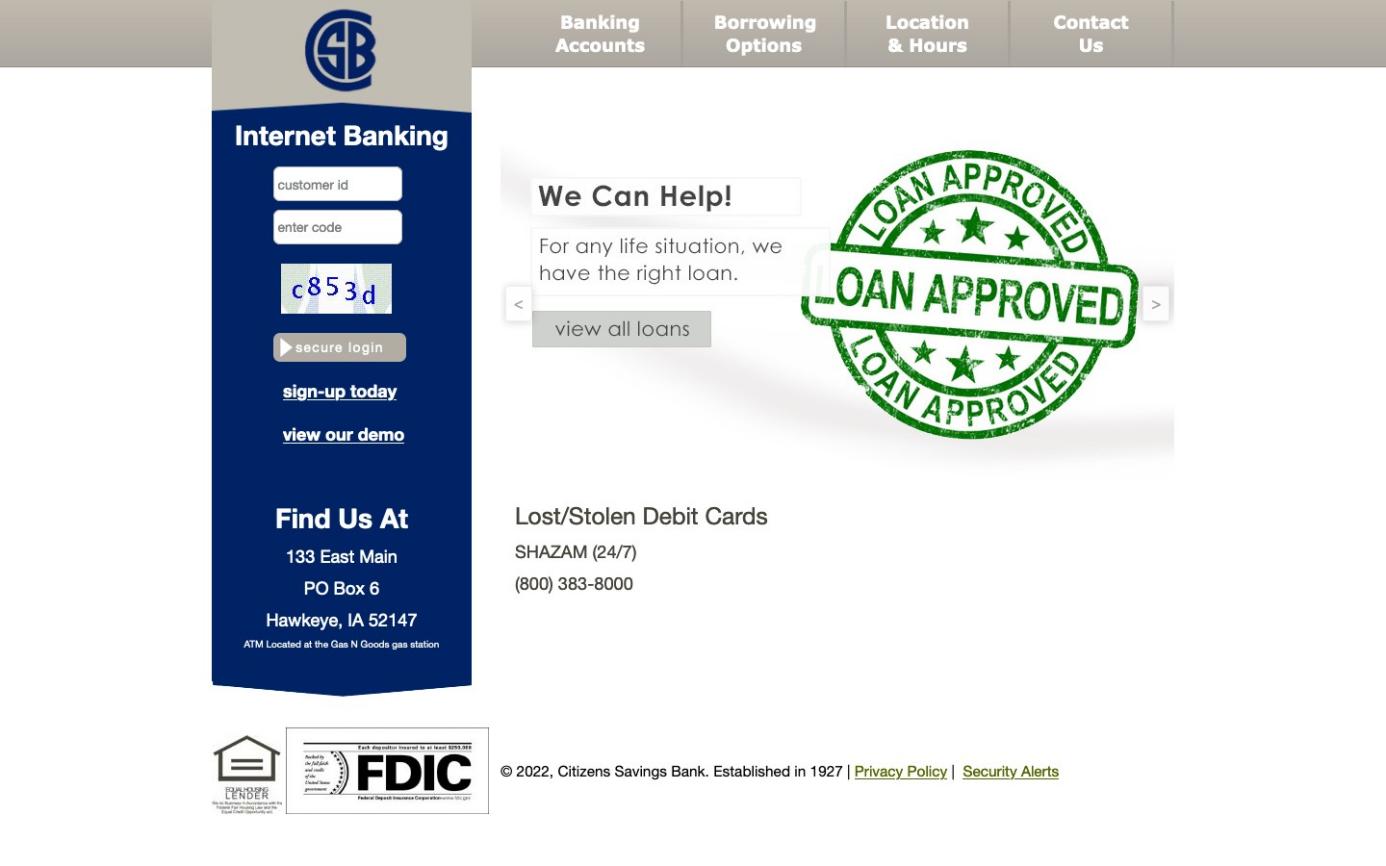


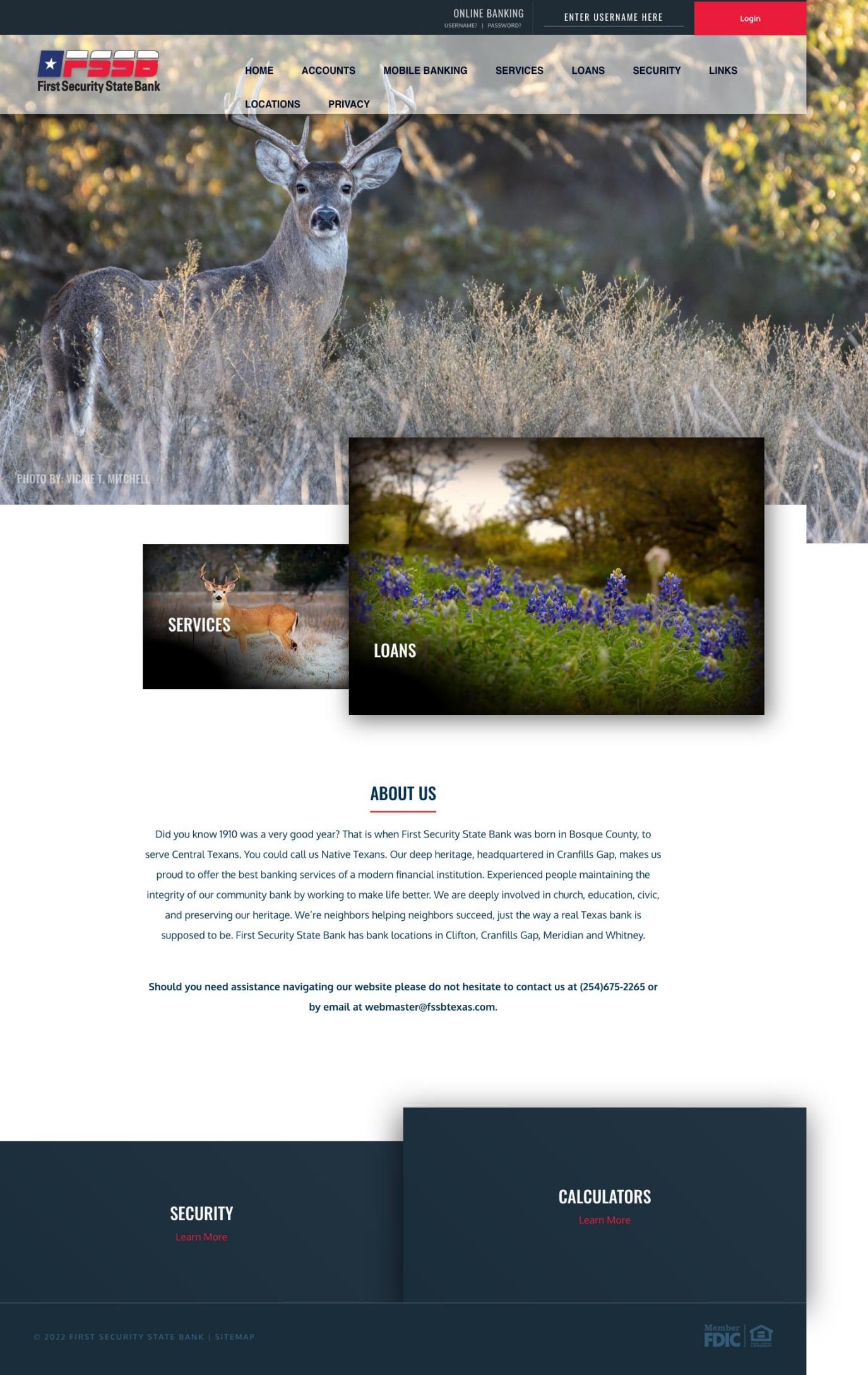


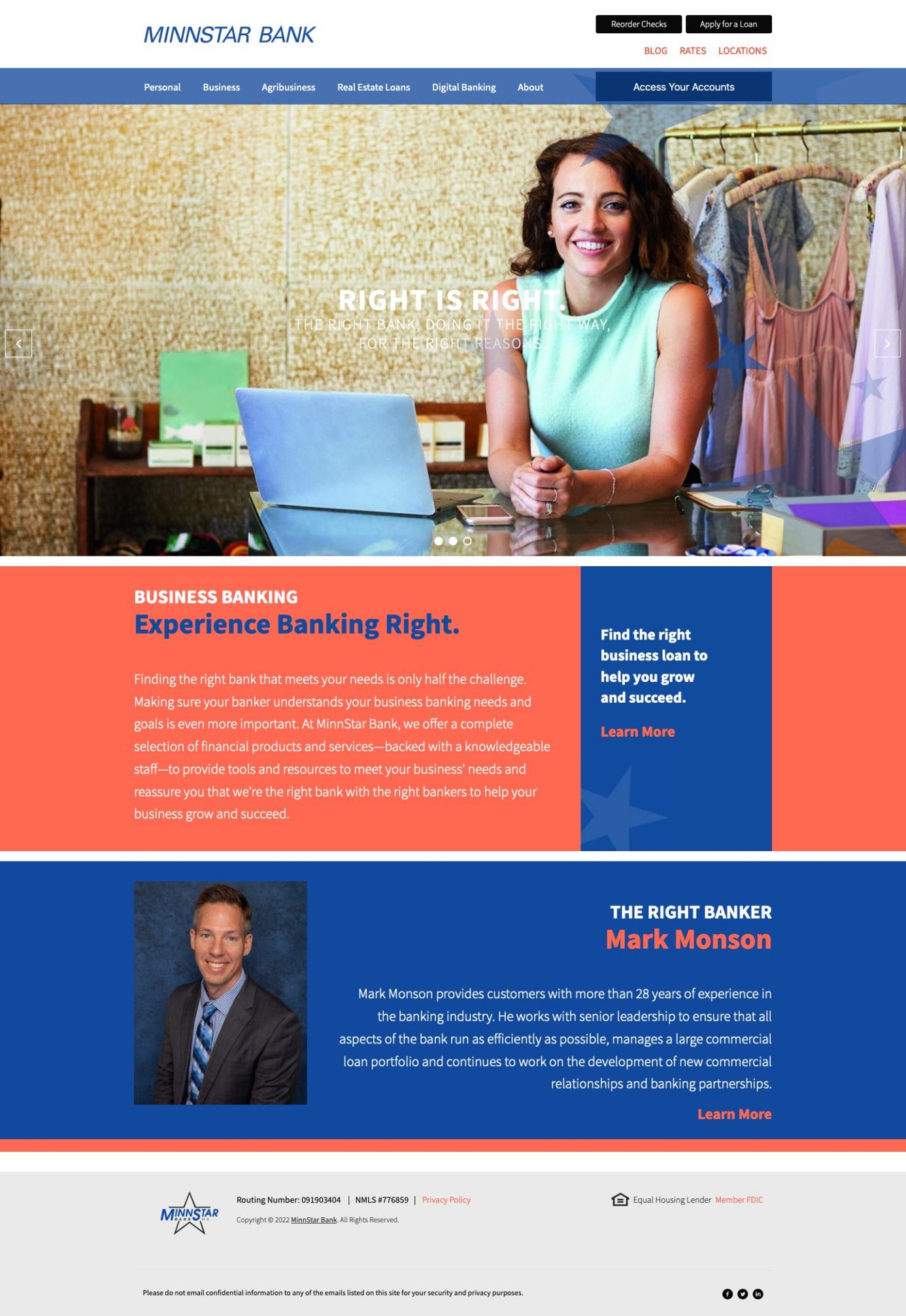


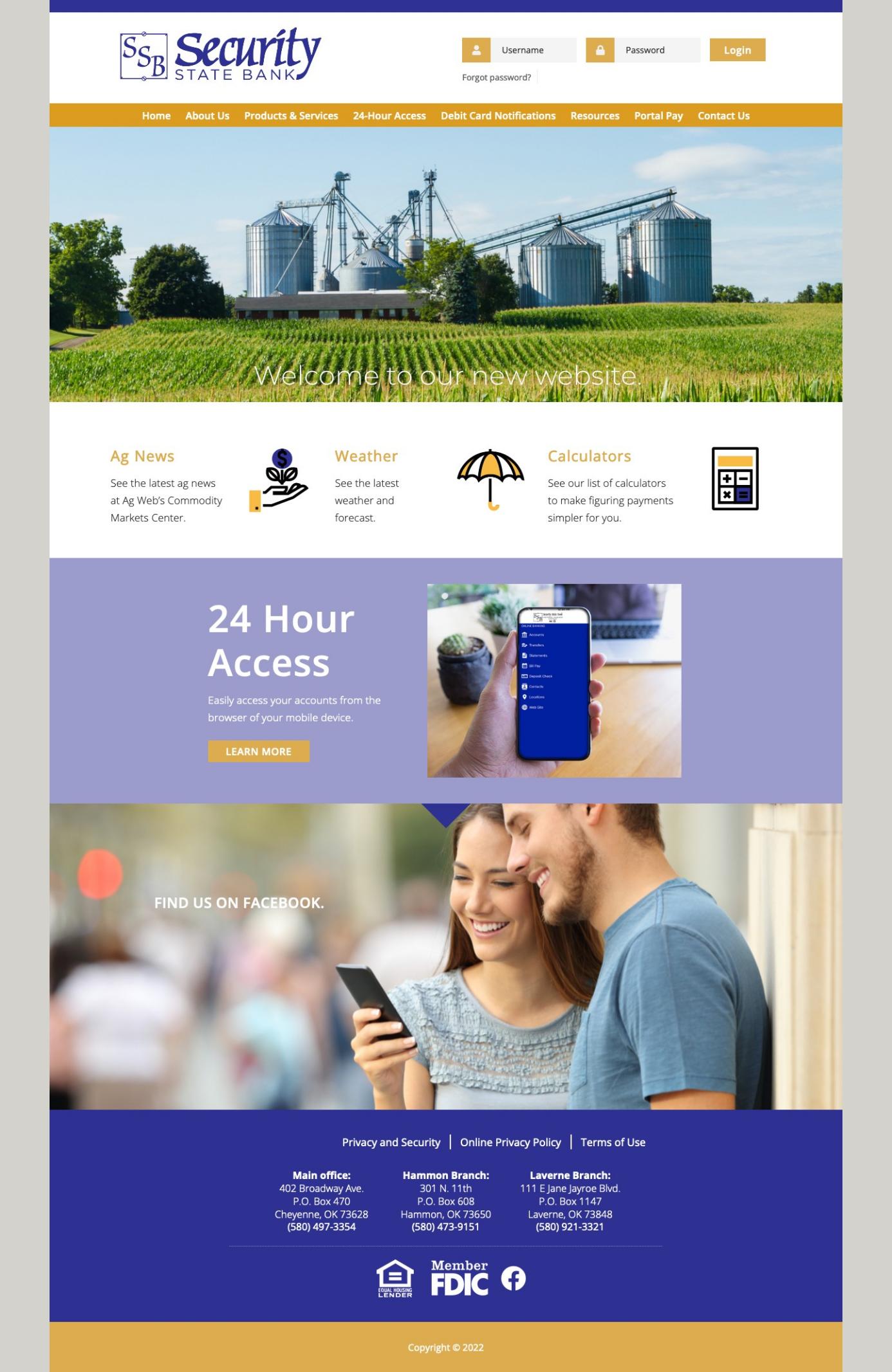


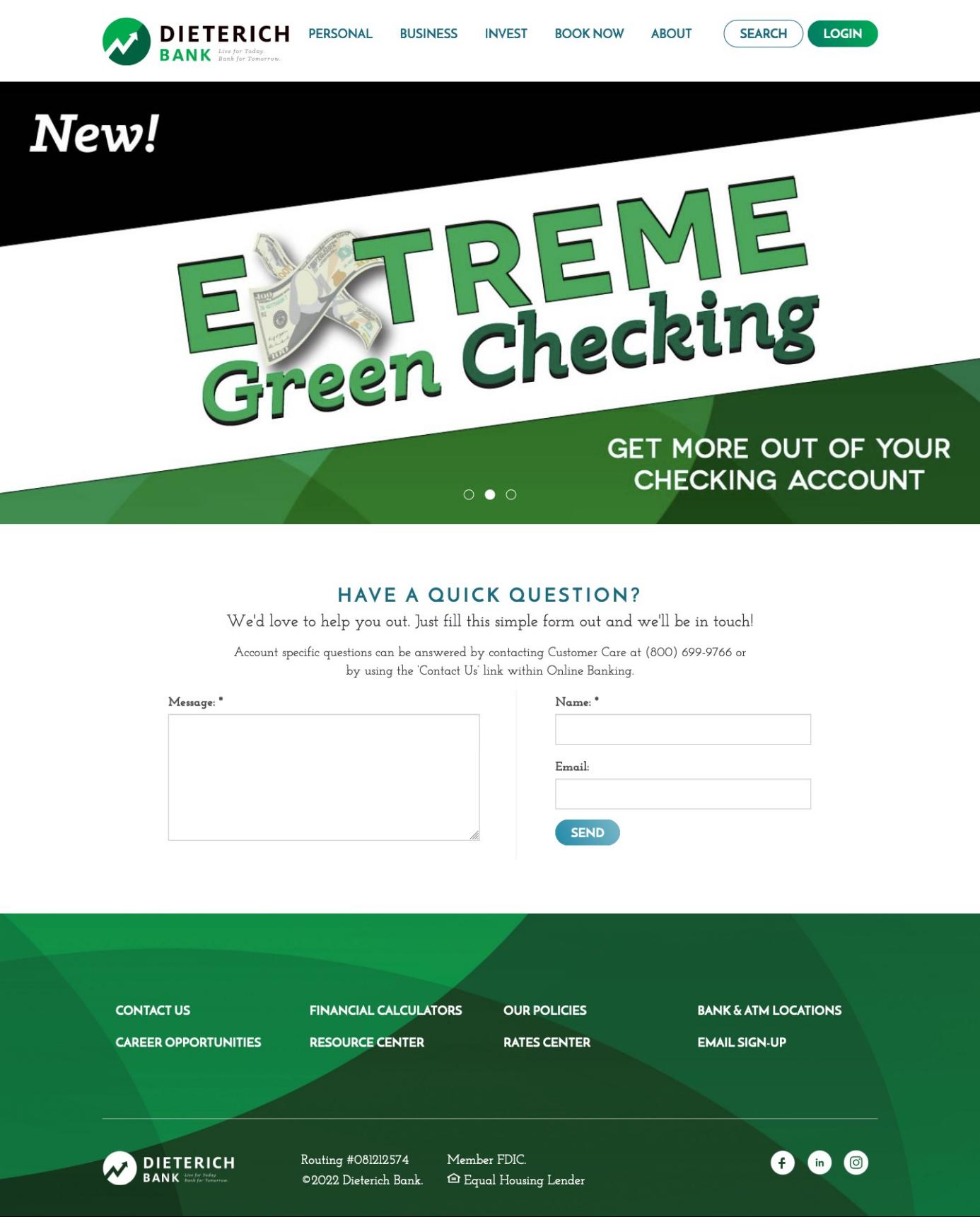


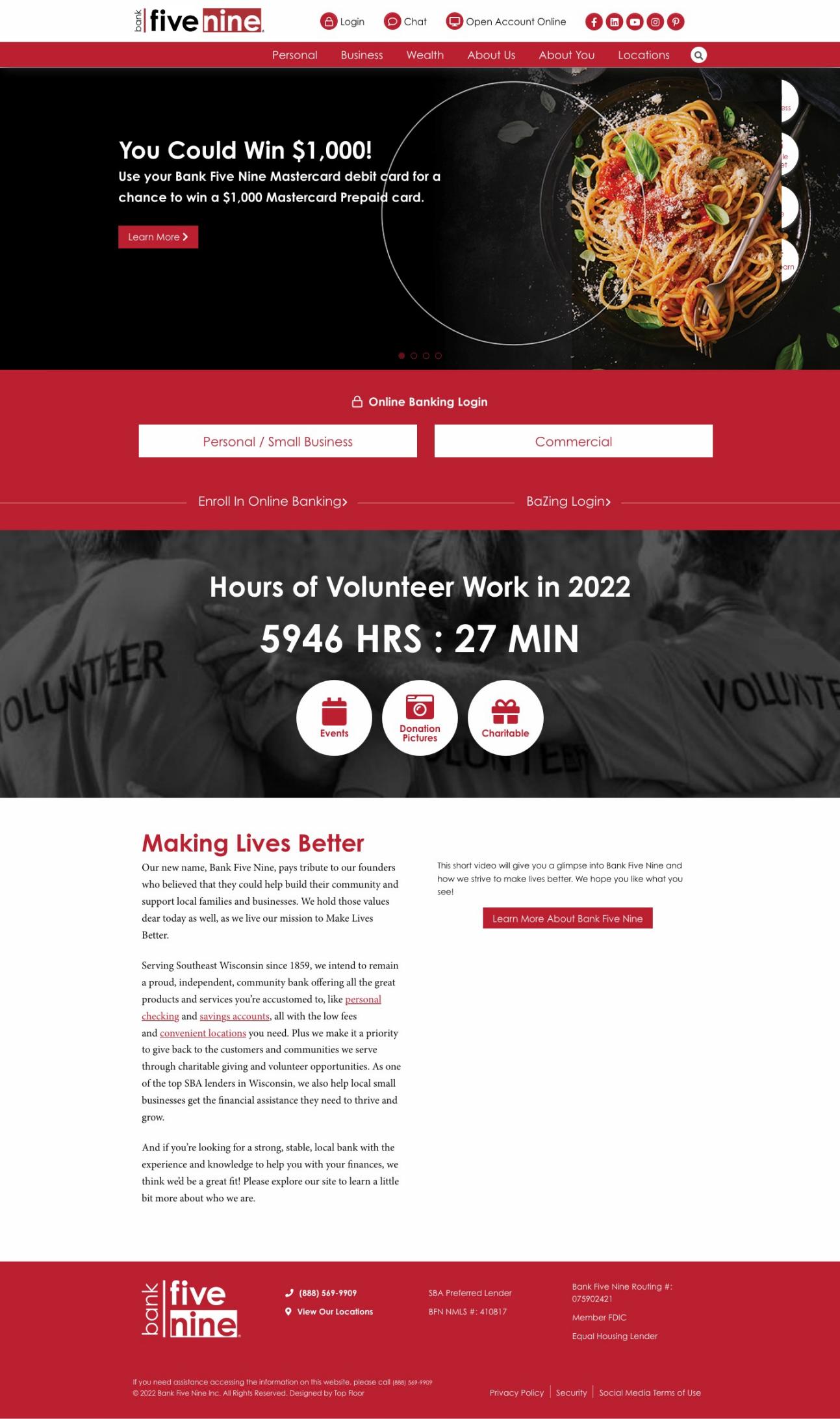


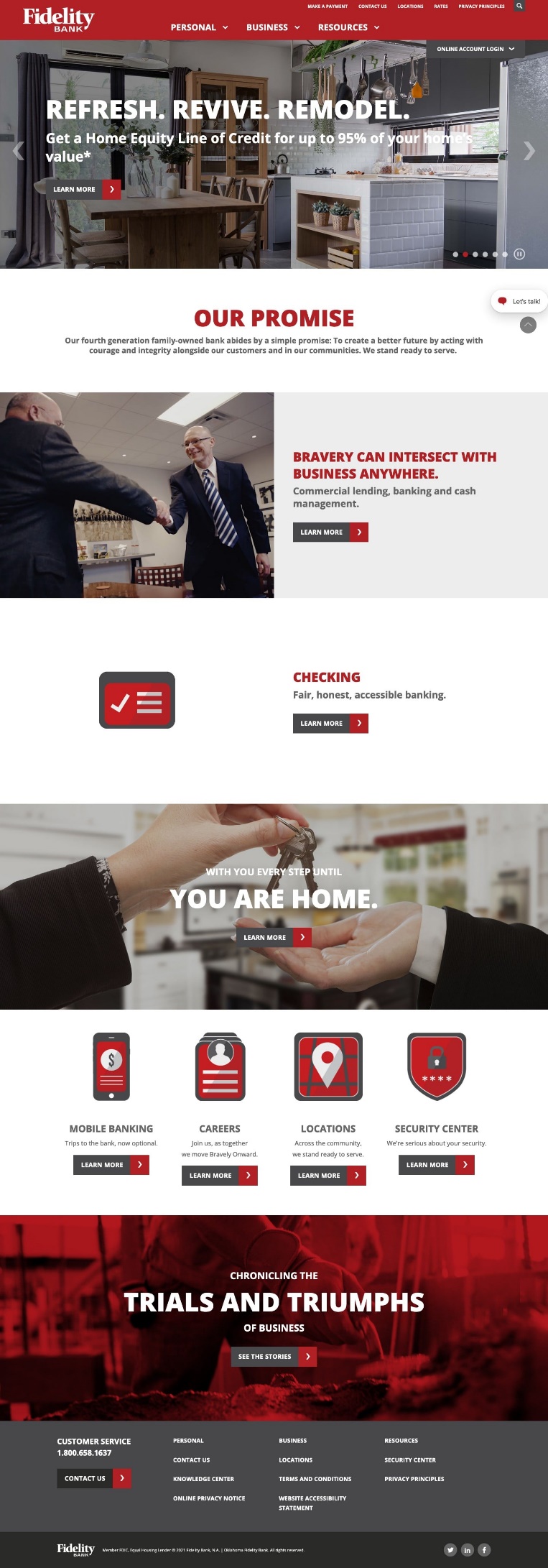


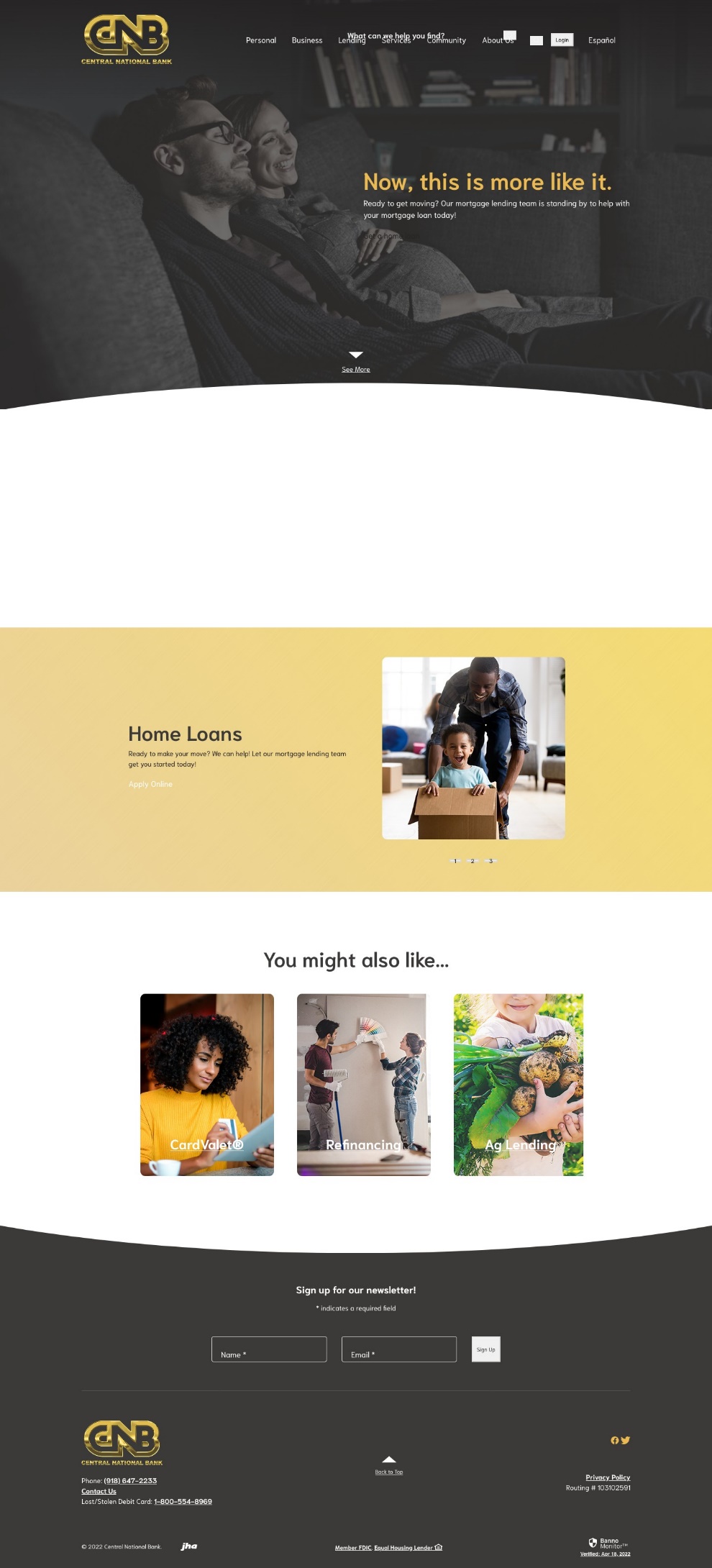


## Messages

### Eight Employer Reviews

| Statement | Message Strength |
| --- | --- |
| The wife of the neighbor of a good friend of mine works in this bank. According to my friend, she always looks very happy, which must be because she likes working at bank [Name]. On the way from my flat to this bank there is a duck pond. I like ducks and would be happy to see them every day if I worked at this bank. | Low |
| According to my neighbor, this bank is a good employer. Once a week, a food truck stops right in front of the door, which I would like to try out, and I generally like to spend my breaks outdoors. I have known the manager of this bank for a long time because my father and he play golf together and he has been to our house for dinner several times. | Low |
| This bank was recommended to me by a distant acquaintance, and I passed it on the bus the other day. I think it could be a good employer for me because the facades are glazed all around, which I always like in buildings. My favorite café is just around the corner, so I can grab a fresh, delicious coffee on my way to work. | Low |
| I found out about this bank through social media and think it must be a very reliable employer. My friend's neighbor worked there for a year and found the furniture in the entrance area very appealing. Also, from the roof of the building you have a breathtaking view over the whole city. I could imagine working here and spending my lunch break up there. | Low |
| I really enjoy working for this bank. I was convinced by the good working atmosphere that’s not only within the department but also with the higher management levels. Employees all benefit from free health insurance, as well as training opportunities and the possibility to use the in-house childcare. All employees have the same opportunities and are treated equally. I can't imagine a better employer. | High |
| This bank is the perfect employer for me because it takes care of not only its customers but also its employees. For example, it has perqs like health insurance, training paid by the company and childcare. Also, the contact with the management is very straightforward, and you have a good chance to move up within the first two years. I can't imagine working for another employer that offers the same benefits and is so renowned. | High |
| This bank has helped me, as an employee, to develop and expand my skills. Every employee has the opportunity to attend training, which the bank funds. Other benefits include sports facilities, daycare and free snacks for all employees. The company is centrally located, so it is easy to reach by public transport or car. | High |
| This bank was a great employer. I worked there for twenty years until I took my well-deserved retirement last year. I have witnessed some changes in the company structure and can say that everything was always well coordinated, and we employees were always informed about the next steps. This transparent and open corporate culture is great. I think this bank is a good place to work for both newcomers and old hands. | High |

### Eight About Us Statements

| Statement | Message Strength |
| --- | --- |
| [Company Name] is very successful, and many people recommend it. It is customer-oriented, and the staff are happy to talk to you. The architecture of the buildings is beautiful, and all the rooms are tastefully decorated and furnished. Ask your grandmother or grandfather, and they will confirm that [Company Name] has been reliable over the years. You can trust this bank, as many investors do. | Low |
| Our founder liked the outdoors and started this brand many years ago to share his passion for fashion and nature with the world. We’re serious about making sure every person in our global supply chain is safe at work and has their rights respected and protected. We set high ethical standards and support our suppliers to help them meet them. Check out our website to learn more about us and browse our range of natural products. | Low |
| On January 27, 1930, the [Name] Banking Company commenced operations with [Person X] as president of the new bank. As the owners did not want to sell the old bank building, the [Family X]s decided to move their bank to a new location in 1934. Mr. [Person Y] was elected President in January 1949 and ran the bank for many years until his death. [Person Z] became President of [Name] Banking in 2010 and still holds the position today. | Low |
| [Company Name]’s goals is to enrich the lives of the people we serve by providing a deeper understanding of banking, business and beyond. Our name comes from our key strategic partner, [Another Name] Companies, and together we offer a comprehensive range of services for you, your life and your business. We hope you like our green and yellow uniforms, which symbolize success and hope. | Low |
| [Company Name] is committed to making communities stronger and financial lives better through responsible growth and empowering those we serve. [Company Name] was founded in 1903, with deposits of $3,338.22. Today, we have $27.2 billion in total assets and more than 2,800 employees in six states. We have the strength of one of America's leading banks, but we still pride ourselves on being a community bank that works hard to make our customers’ dreams come true. | High |
| With 23 branches throughout [Region], [Company Name] is one of the leading private banks in the region. Founded in 1992, [Company Name] has served its clients for more than 30 years with high levels of expertise. We provide flexible, efficient and personalized services and have strong partnerships with international organizations that enable us to offer long-term foreign currency financing to our clients. | High |
| Consumer and commercial banking products and services are offered through [Company Name]. [Company Name]’s goal is to help people to reach their full financial potential, whether our clients, our team members, or the communities we serve. This approach has served us well, as today X is a high-performing midcap bank with more than $57 billion in total assets and $7.3 billion in capital. In fact, Forbes named us one of America's Best Banks. | High |
| [Company Name] is a community bank dedicated to enriching the communities we serve. Our professional bankers are fully experienced in retail, commercial and real estate banking and are committed to providing high-quality, efficient financial services. We fulfill our mission by continually improving our customer service and response, seeking new investment and service opportunities in our communities, and maintaining a strong and profitable bank. | High |

## Screenshots of All Workload Tasks


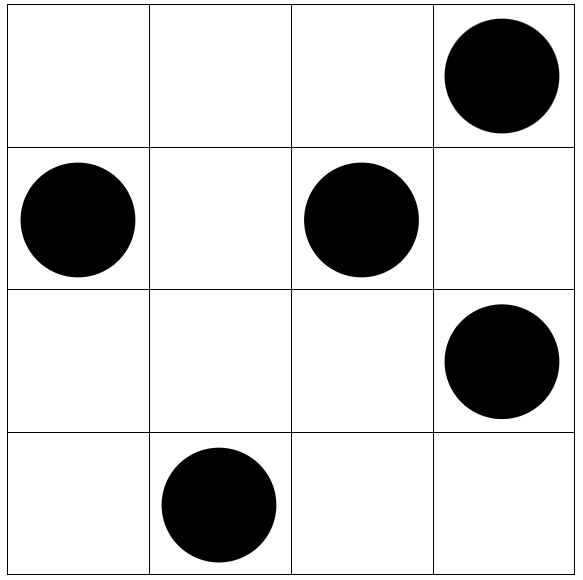

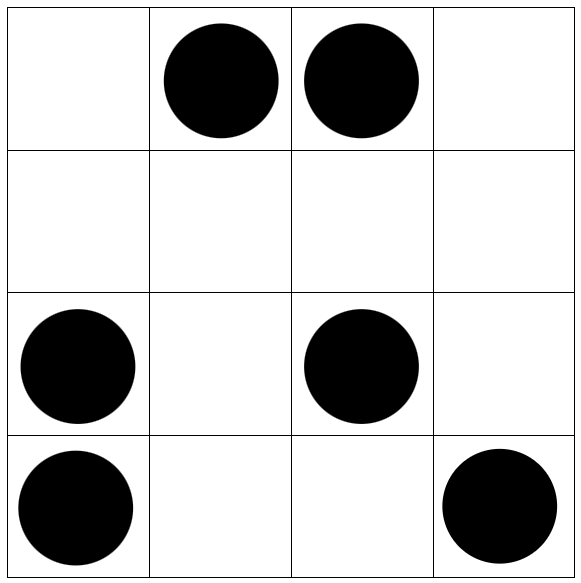


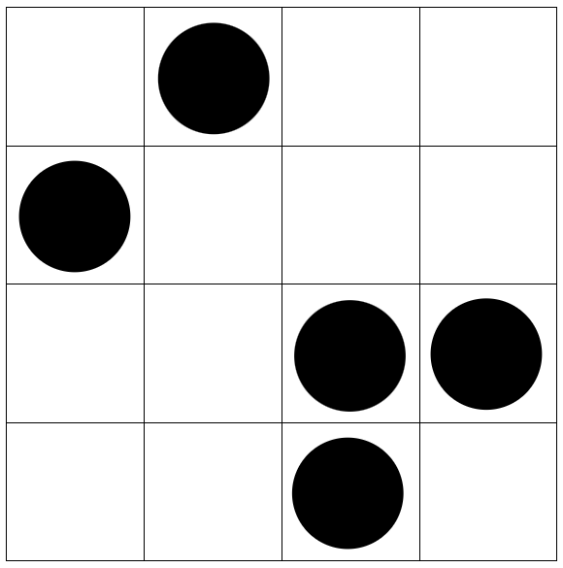

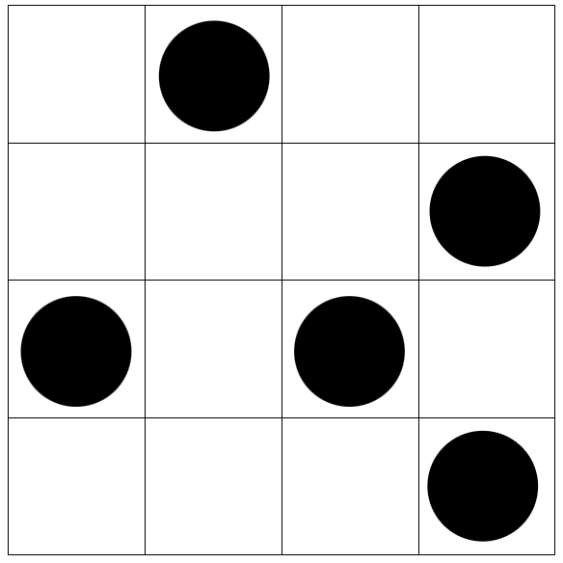


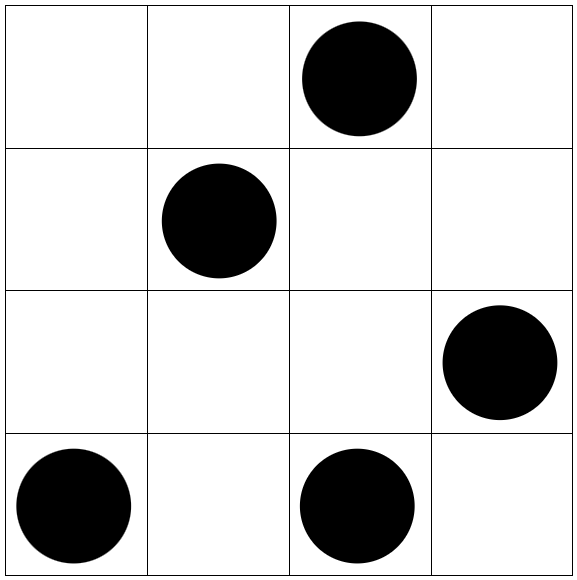

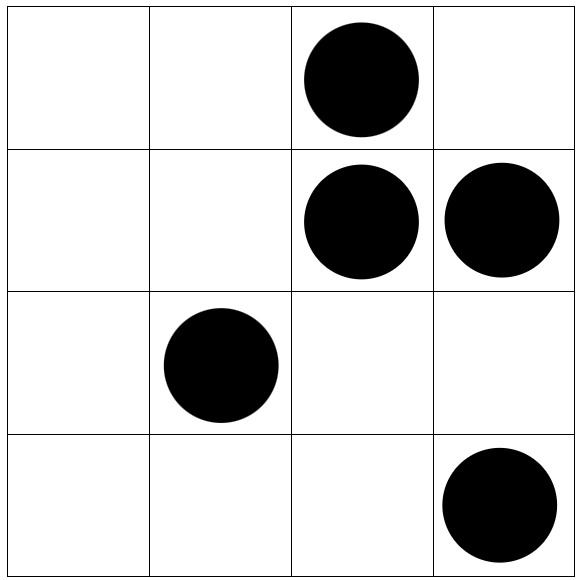


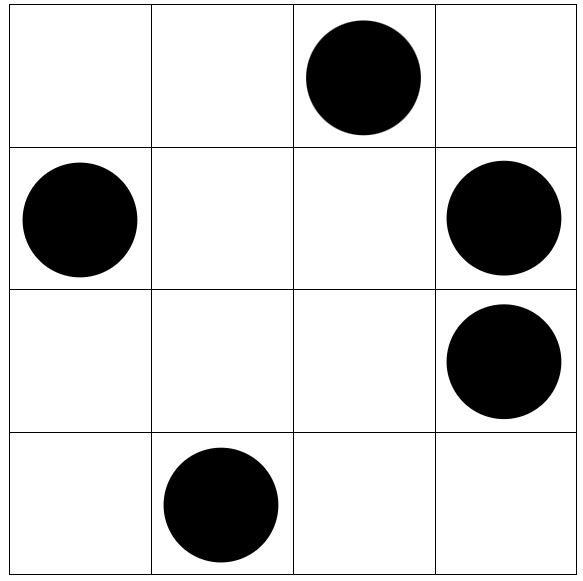

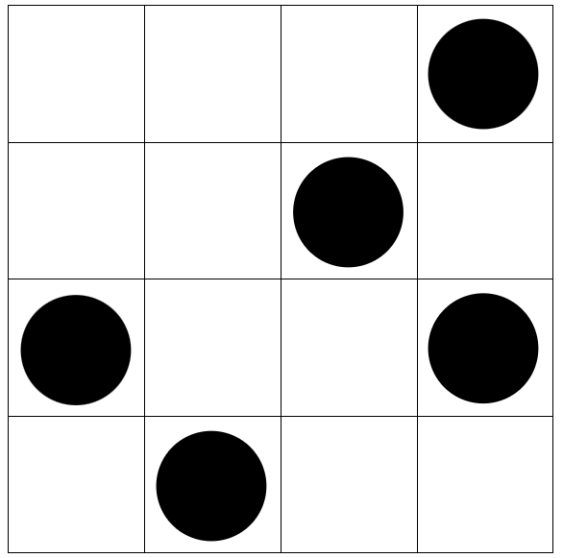


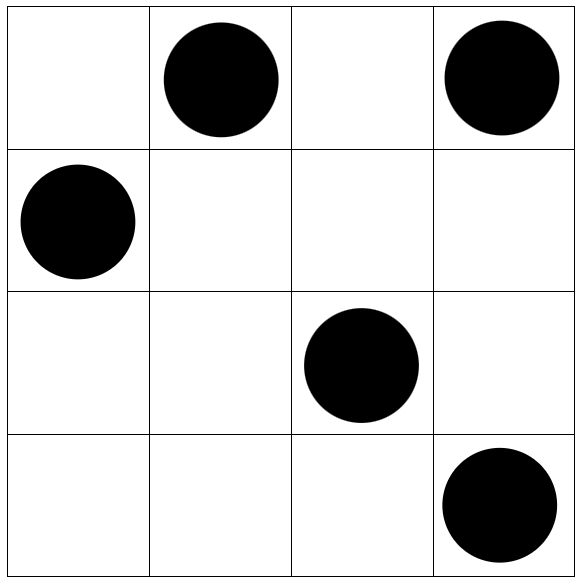

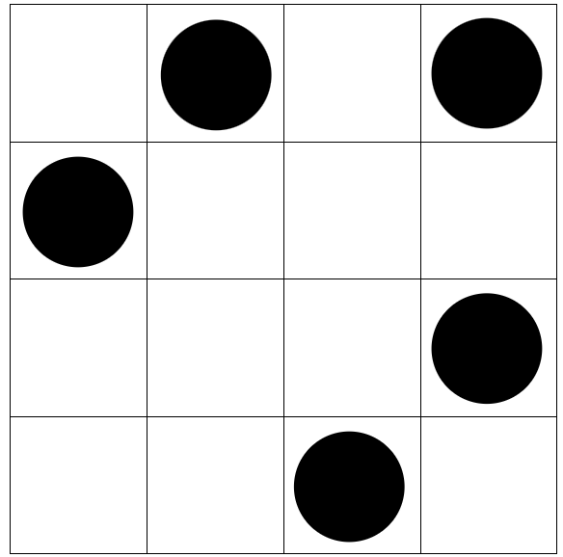


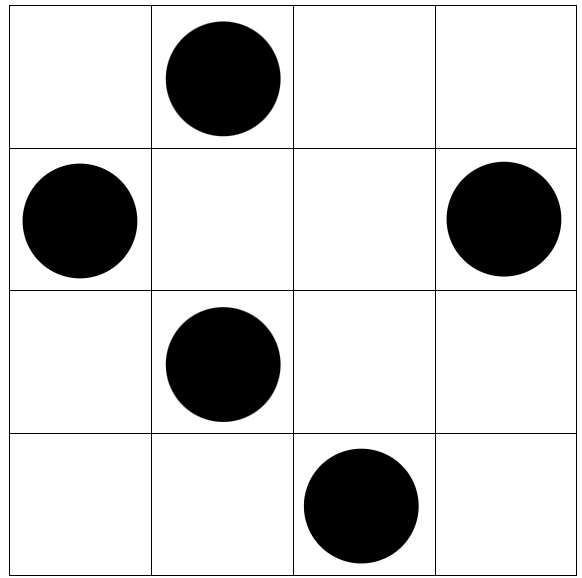

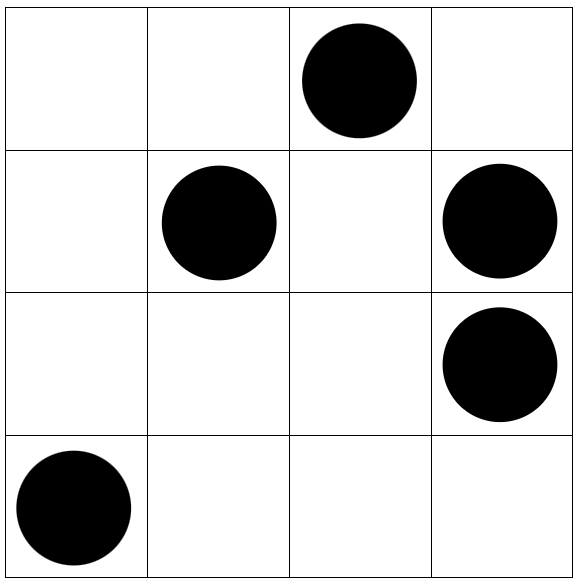


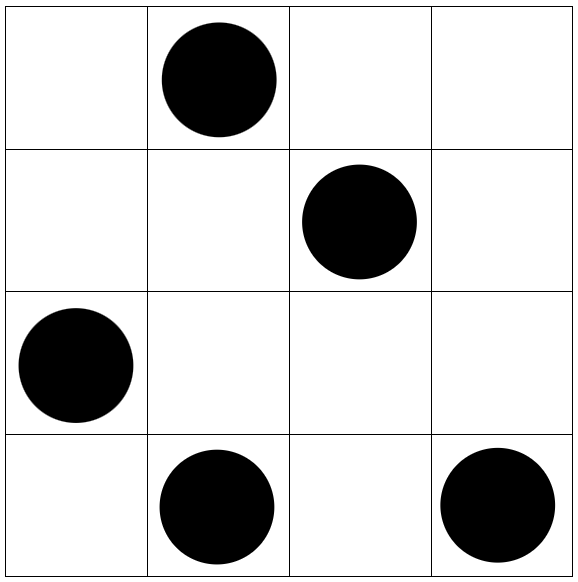

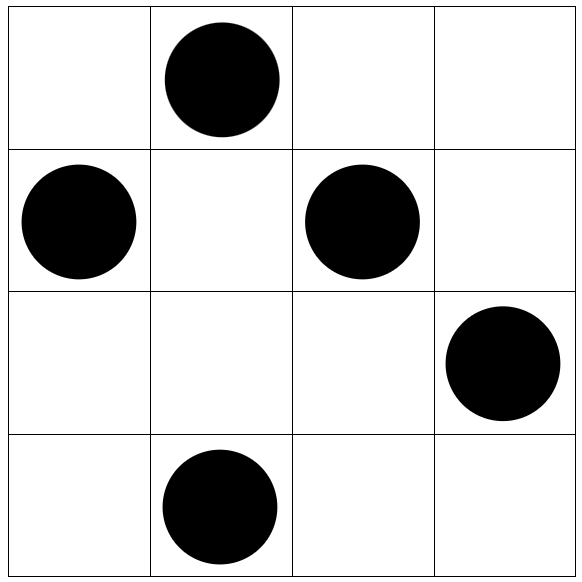


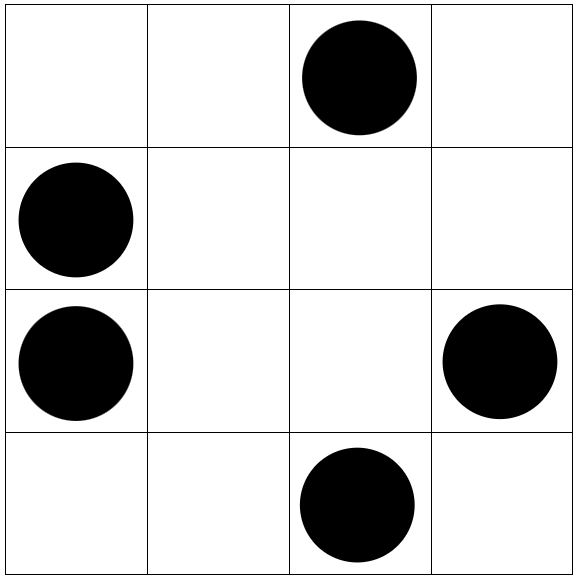

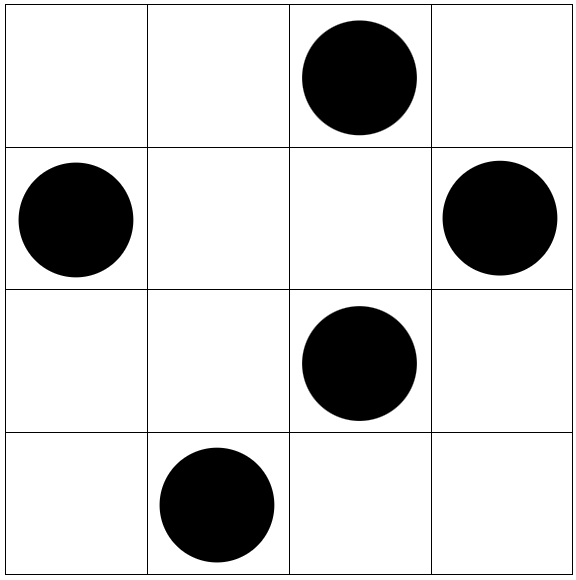


# References

Ageeva, E., Melewar, T. C., Foroudi, P., Dennis, C., & Jin, Z. (2018). Examining the influence of corporate website favorability on corporate image and corporate reputation: Findings from fsQCA. *Journal of Business Research*, *89*, 287-304. <https://doi.org/10.1016/j.jbusres.2018.01.036>

Brengman, M., & Karimov, F. P. (2012). The Effect of Web Communities on Consumers' Initial Trust in B2C E‐Commerce Websites. *Management Research Review*, *35*(9), 791-817. <https://doi.org/10.1108/01409171211256569>

Chmielewski, M., & Kucker, S. C. (2020). An MTurk crisis? Shifts in data quality and the impact on study results. *Social Psychological and Personality Science*, *11*(4), 464-473. <https://doi.org/10.1177/1948550619875149>

Kennedy, R., Clifford, S., Burleigh, T., Waggoner, P. D., Jewell, R., & Winter, N. J. (2020). The shape of and solutions to the MTurk quality crisis. *Political Science Research and Methods*, *8*(4), 614-629. <https://doi.org/10.1017/psrm.2020.6>

Loken, B., & Ward, J. (1990). Alternative approaches to understanding the determinants of typicality. *Journal of Consumer Research*, *17*(2), 111-126. <https://doi.org/10.1086/208542>

Miniukovich, A., & Marchese, M. (2020). Relationship Between Visual Complexity and Aesthetics of Webpages. Proceedings of the 2020 CHI Conference on Human Factors in Computing Systems,

San José Cabezudo, R., Gutiérrez-Arranz, A. M., & Gutiérrez-Cillán, J. (2009). The combined influence of central and peripheral routes in the online persuasion process. *Cyberpsychology & behavior*, *12*(3), 299-308. <https://doi.org/10.1089/cpb.2008.0188>
